# Light‐Activatable MBD‐Readers of 5‐Methylcytosine Reveal Domain‐Dependent Chromatin Association Kinetics In Vivo

**DOI:** 10.1002/advs.202307930

**Published:** 2024-01-02

**Authors:** Tzu‐Chen Lin, Lena Engelhard, Benedikt Söldner, Rasmus Linser, Daniel Summerer

**Affiliations:** ^1^ Department of Chemistry and Chemical Biology TU Dortmund University Otto‐Hahn Str. 4a 44227 Dortmund Germany

**Keywords:** DNA methylation, epigenetics, genetic code expansion, methyl‐CpG‐binding domains, optochemical biology

## Abstract

5‐Methylcytosine (5mC) is the central epigenetic mark of mammalian DNA, and plays fundamental roles in chromatin regulation. 5mC is dynamically read and translated into regulatory outputs by methyl‐CpG‐binding domain (MBD) proteins. These multidomain readers recognize 5mC via an MBD domain, and undergo additional domain‐dependent interactions with multiple additional chromatin components. However, studying this dynamic process is limited by a lack of methods to conditionally control the 5mC affinity of MBD readers in cells. Light‐control of MBD association to chromatin by genetically encoding a photocaged serine at the MBD‐DNA interface is reported. The authors study the association of MBD1 to mouse pericentromeres, dependent on its CxxC3 and transcriptional repressor domains (TRD) which interact with unmethylated CpG and heterochromatin‐associated complexes, respectively. Both domains significantly modulate association kinetics, arguing for a model in which the CxxC3 delays methylation responses of MBD1 by holding it at unmethylated loci, whereas the TRD promotes responses by aiding heterochromatin association is studied. Their approach offers otherwise inaccessible kinetic insights into the domain‐specific regulation of a central MBD reader, and sets the basis for further unravelling how the integration of MBDs into complex heterochromatin interaction networks control the kinetics of 5mC reading and translation into altered chromatin states.

## Graphical abstract

5‐Methylcytosine (5mC) is the central epigenetic mark of mammalian DNA, and plays important roles in chromatin regulation. The authors report light‐control of a methyl‐CpG‐binding domain, the reader of 5mC, by genetic encoding of a photocaged serine at the MBD‐DNA interface. Kinetic studies of MBD1 association to pericentromeric chromocenters in mouse fibroblasts reveal a role of two additional domains of MBD1 in regulating this association as initial step that precedes the translation of 5mC into altered regulatory states in mammalian chromatin.

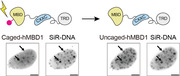

## 1 Introduction

**Figure 1:**
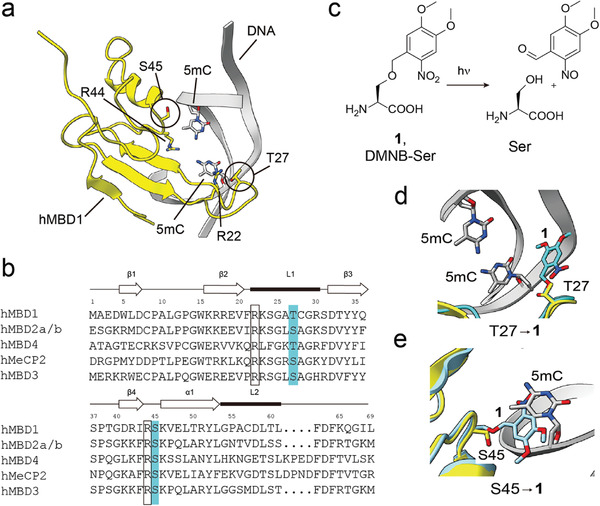
Light control of MBD1 5mCpG affinity using a genetically encoded, photocaged serine. a) Solution NMR structure of hMBD1 in complex with 5mCpG‐containing DNA duplex (PDB 1IG4^[^ ^8^ ^]^). b) Sequence alignment of human MBD family proteins. Residues aligned with hMBD1 T27 and S45 marked in cyan. R22 and R44 recognize 5mCpG guanines (not shown), and are critical for affinity. c) Light deprotection of photocaged serine **1**. d,e) Energy minimized structures of d) hMBD1‐T27→**1** (cyan) and e) hMBD1‐S45→**1** (cyan), each superimposed with the solution NMR structure of hMBD1‐DNA complex (yellow; PDB 1IG4^[^ ^8^ ^]^).

5‐Methylcytosine (5mC, **Figure** **1**a) is a dynamic regulatory element of mammalian genomes with key roles in transcription regulation, cell differentiation, development, and cancer. 5mC is written by DNA‐methyltransferases (DNMT) mainly at CpG dyads, and serves as repressive mark mainly associated with silenced chromatin.^[^ ^1^ ^]^ Methyl‐CpG‐binding domain (MBD) proteins are the readers of 5mC that dynamically coordinate the crosstalk between 5mC, repressive histone modifications such as H3K9, and other regulatory elements. The MBD core family proteins (comprising MBD1, MBD2, MBD3, MBD4, and MeCP2) share a conserved 70–85 amino acid MBD domain recognizing methylated CpGs (5mCpGs; MBD3 contains a dysfunctional MBD). In contrast, the individual proteins differ in additional domains that equip them with further interaction surfaces and catalytic activities, and thus with distinct regulatory functions.^[^ ^2^ ^]^

The ability to control the 5mC affinity of MBDs with temporal resolution in cells would enable studying how their integration into chromatin interaction networks via additional regulatory domains control their 5mC reading kinetics, which ultimately controls the dynamic 5mC translation into altered chromatin states. However, there are no methods for conditionally controlling MBD‐5mC interactions. Light offers control of protein functions with excellent spatiotemporal resolution and tunability, given the availability of suited photoresponsive tools.^[^ ^3^ ^]^

We report light‐control of MBDs by caging the 5mCpG interface via a genetically encoded^[^ ^4^ ^]^ photocaged^[^ ^5^ ^]^ serine, enabling the expression of transiently inactive MBD proteins, and their rapid activation with a light pulse. We study the domain‐dependence of MBD1‐association kinetics to pericentromeric DNA in mouse fibroblasts. MBD1 is the largest MBD protein and contains additional domains,^[^ ^2^ ^]^ two of which having affinity to either unmethylated DNA (CxxC3 domain^[^ ^6^ ^]^) or heterochromatin‐associated factors (transcriptional repressor domain; TRD^[^ ^7^ ^]^). This integration into larger chromatin interaction networks may modulate the 5mC association kinetics of MBD1 as the initial step for controlling the dynamics of 5mC regulatory outputs.

## 2 Results and Discussion

We initially identified promising residues at the MBD1‐DNA interface (Figure 1a) for caging, focusing on residues conserved in core family MBDs to ensure potential generalization (Figure 1b). We envisaged a replacement with 4,5‐dimethoxy‐2‐nitrobenzyl‐L‐serine **1** (Figure 1c) that can be incorporated into proteins in response to the amber codon (TAG) in mammalian cells via an engineered *Escherichia coli* leucyl‐tRNA‐synthetase (LRS)/tRNA^Leu^ amber suppressor pair. ^[^ ^5^, ^9^ ^]^ Light‐irradiation of **1** allows effective, scarless decaging to the natural serine residue. Molecular modelling studies using GROMACS^[^ ^10^ ^]^ based on an MBD1 NMR solution structure^[^ ^8^ ^]^ suggested T27 and S45 as promising candidates (Figure 1a,b; T27 is replaced by a serine in MBD2‐3 and MeCP2). Both sites are located close to one strand of the bound DNA duplex, and the models suggested that the caging group of **1** would effectively clash with the DNA backbone or one of the 5mC nucleobases of the target CpG dinucleotide, respectively (Figure 1d,e).

**Figure 2:**
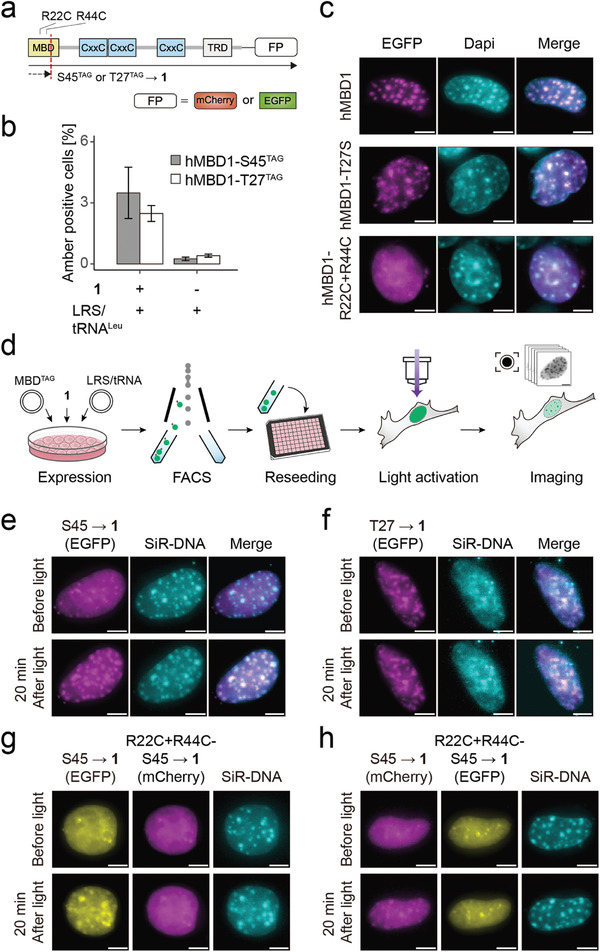
Light control of the hMBD1‐5mCpG interface. a) Domain structure of hMBD1 constructs (FP = fluorescent protein). Full‐length protein is only expressed after suppression of amber codons (dashed red line) with **1**. b) Incorporation fidelity of **1** at hMBD1 amber codons in HEK293T cells co‐expressing the LRS/tRNA^Leu^ pair measured by FACS (two independent biological experiments). c) Localization of wt. hMBD1, hMBD1‐T27S, and hMBD1‐R22C+R44C in NIH/3T3 cells. Dapi staining indicates chromocenters. Scale bar: 5 µm. See Tables S4,S5 (Supporting Information) for Pearson's correlation coefficient and foci number quantification, respectively. d) Workflow of light activation experiments. e,f) Light activation of EGFP‐tagged e) hMBD1‐S45→**1** and f) hMBD1‐T27→**1** in NIH/3T3 cells. SiR‐DNA stain indicates chromocenters. Scale bar: 5 µm. See Tables S4,S5 (Supporting Information) for Pearson's correlation coefficient and foci number quantification, respectively. g,h) Co‐expression and simultaneous light activation of g) EGFP‐tagged hMBD1‐S45→**1** and mCherry‐tagged hMBD1‐R22C+R44C‐S45→**1** or h) mCherry‐tagged hMBD1‐S45→**1** and EGFP‐tagged hMBD1‐R22C+R44C‐S45→**1** in NIH/3T3 cells. SiR‐DNA stain indicates chromocenters. Scale bar: 5 µm. See Tables S4,S5 (Supporting Information) for Pearson's correlation coefficient and foci number quantification, respectively.

We constructed vectors encoding full‐length human MBD1 (hMBD1) containing a single in‐frame amber codon (TAG) at S45 or T27 in the MBD domain, and a C‐terminal EGFP tag for monitoring incorporation of **1** by fluorescence (**Figure** **2**a). Co‐expression of the LRS/tRNA^Leu^ pair in HEK293T cells in the presence of 0.05 mm **1** resulted in increased EGFP fluorescence compared to cells grown in the absence of **1**, indicating its faithful incorporation at both S45 and T27 (Figure 2b; Figure S1, Supporting Information).

In mouse fibroblast NIH/3T3 cells, pericentromeric heterochromatin enriched with 5mCpGs accumulates wild‐type hMBD1, leading to the formation of chromocenters that can be imaged using DNA stains or fluorescently labeled MBDs^[^ ^11^ ^]^ (Figure 2c). We first ensured that a T27→S mutation in the MBD domain (occurring after hMBD1 S27→**1** decaging) does not influence hMBD1´s cellular localization. Indeed, EGFP‐labeled wt. or T27→S hMBD1 expressed in NIH/3T3 cells colocalized with Dapi at chromocenters. In contrast, a R22C+R44C control mutant lacking critical interactions with the 5mCpG guanines (Figure 1a,b) with an associated loss of affinity^[^ ^8^, ^12^ ^]^ did not (Figure 2c). Moreover, wt. and S27→T hMBD1 perfectly colocalized in co‐expression experiments (Figure S2, Supporting Information). We next co‐expressed the LRS/tRNA^Leu^ pair with either the hMBD1‐S45→TAG or the hMBD1‐T27→TAG amber mutant construct in presence of **1**. After 21 h of incubation, we removed **1** from the culture to terminate hMBD1 expression, and isolated cells that had expressed full‐length hMBD1 constructs by fluorescence‐activated cell sorting (FACS, Figure 2d). In imaging, hMBD1‐S45→**1** did not colocalize with the DNA stain SiR‐DNA, but rather resembled the phenotype of the hMBD1‐R22C+R44C mutant (Figure 2e, “before light”), indicating that **1** indeed abolished pericentromer binding. In contrast, hMBD1‐T27→**1** colocalized with SiR‐DNA at chromocenters (Figure 2f “before light”), indicating no effect of **1** on 5mCpG‐binding when present at position T27.

We next uncaged hMBD1 proteins by exposing the cells to a short light pulse (395/25 nm LED, 50 ms; Figure S3 (Supporting Information) shows in vitro uncaging efficiency of **1**; Figure S4 (Supporting Information) shows in cellulo light dosage experiments). Whereas hMBD1‐S45→**1** was localized at chromocenters within 20 min after light, hMBD1‐T27→**1** was not (Figure 2e,f; Figures S6,S7, Supporting Information). To test if this localization change was due to decaging of the 5mC‐MBD interfaces or to another cellular response to the uncaging process, we co‐expressed hMBD1‐S45→**1** and the non‐5mCpG binding hMBD1‐R22C+R44C‐S45→**1** mutant fused to EGFP and mCherry, respectively. Both proteins did not localize to chromocenters before light, and only hMBD1‐S45→**1** showed the expected localization change after light (Figure 2g). We further made the same observation with identical proteins but with switched fluorophores (Figure 2h). These results collectively indicate that the observed chromocenter association of hMBD1 is due to a light‐dependent activation of its 5mCpG interaction surface, and not due to other effects of the uncaging process. Furthermore, we characterized the binding ability of hMBD1‐S45→**1** isolated from irradiated or nonirradiated cells to DNA by in vitro pulldown assays. While both wt. hMBD1 and the irradiated hMBD1 S45→**1** bound to a methylated (but not unmethylated) 200 nt DNA probe containing part of the human VEGF‐A promoter, the nonirradiated hMBD1 S45→**1** did not bind DNA at all (Figure S5, Supporting Information). This provides additional evidence that the chromocenter association is due to the activation of the hMBD1‐5mCpG interaction surface by light decaging of **1**.

**Figure 3:**
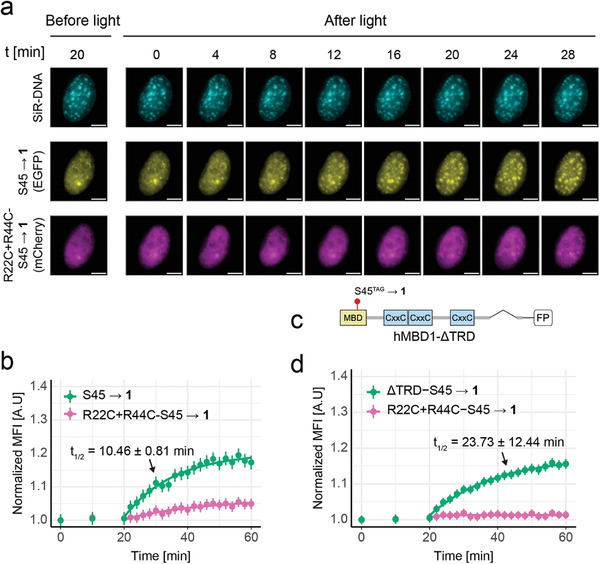
Studying hMBD1 chromocenter association kinetics in NIH/3T3 cells by light activation. a) Time‐resolved monitoring of chromocenter association before and after simultaneous light activation of co‐expressed hMBD1‐S45→**1** and hMBD1‐R22C+R44C‐S45→**1**. Chromocenters are indicated by SiR‐DNA stain. Scale bar: 5 µm. b) Time courses and fitting curves of experiment conducted as in Figure 3a (N = 163 chromocenters, 6 cells; *t*_1/2_ from three biological replicates totalling 530 chromocenters of 25 cells. c) Domain structure of hMBD1‐S45→**1**_ΔTRD mutant. d) Time courses and fitting curves of experiment with hMBD1‐ΔTRD‐S45→**1** (N = 275 chromocenters, 12 cells; *t*_1/2_ from three biological replicates totalling 894 chromocenters of 40 cells).

We next evaluated if our new tool would enable the precise characterization of hMBD1 association kinetics to genomic 5mCpG targets in vivo by time‐resolved measurements. We imaged NIH/3T3 cells co‐expressing differently labeled hMBD1‐S45→**1** and hMBD1‐R22C+R44C‐S45→**1** 20 min before light activation in 10 min intervals, decaged the proteins, and acquired images every 4 min over a 28 min period (**Figure** **3**a, see Movie S1, Supporting Information).

None of the proteins changed localization before light, but hMBD1 rapidly localized to chromocenters after decaging, with a marked increase of chromocenter fluorescence after 8 min that started to plateau after ≈20 min. In contrast, hMBD1‐R22C+R44C‐S45→**1** did not re‐localize in this time window (Figure 3a; Figures S9,S10, Supporting Information).

To obtain quantitative kinetics data, we measured for both fluorophores the mean fluorescence intensities (MFI) of all chromocenter areas in the cells, normalized them first to the mean nuclear background fluorescence of the respective cells, then to the intensity before decaging (i.e., 0 min), and plotted it against the observation time (Figure 3b; Figure S17, Supporting Information). Whereas we observed a rapid increase in chromocenter association after light activation for hMBD1‐S45→**1**, the R22C+R44C mutant showed only a slight background increase (Figure 3b; Figures S9,S10, Supporting Information). We also quantified the number of foci, and the average foci number per cell and plotted them against the observation time (Figure S8, Supporting Information). The kinetic curve obtained by foci quantification aligned with the one of MFI quantification.

Because remaining nuclear fluorescence signal of hMBD1‐S45→**1** after chromocenter saturation indicated an excess of MBD relative to chromocenter 5mCpG binding sites, we used pseudo‐first‐order association kinetics as simplified model for obtaining half‐saturation times *t*_1/2_ via fitting (*y* = *y*_0_ + (*y_max_* − *y*_0_)(1 − *e* ^−*kt*^)). ^[^ ^13^ ^]^ For our competitive conditions in the presence of hMBD1‐R44C+R44C‐S45→**1**, we obtained a *t*_1/2_ = 10.46 ± 0.81 min for hMBD1 S45→. Moreover, we obtained a similar *t*_1/2_ of 10.75 ± 1.35 min for single transfection experiments with EGFP‐labelled hMBD1 S45→**1** (Figure S16, Supporting Information).

We next investigated, if hMBD1 domains with affinity to other chromatin components would modulate this readers chromocenter association kinetics.

hMBD1 comprises two domains with distinct regulatory functions in addition to the MBD domain. Specifically, the TRD of MBD1 has a pivotal function in mediating the repressive output of 5mC,^[^ ^2^ ^]^ e.g., by interacting with the MCAF1/SETDB1 H3K9 writer complex that promotes HP1‐mediated heterochromatin formation^[^ ^7^, ^14^ ^]^, and with the histone deacetylase HDAC3.^[^ ^15^ ^]^ To investigate, how the TRD and its interactions may regulate 5mCpG association kinetics of hMBD1, we co‐expressed the EGFP‐tagged, TRD‐truncated mutant hMBD1‐ΔTRD‐S45→**1** (Figure 3c) with our mCherry‐tagged hMBD1‐R22C+R44C‐S45→**1** control construct in NIH/3T3 cells. After light, we observed a much slower chromocenter association compared to the full‐length wt. hMBD1 (*t*_1/2_ = 23.73 ± 12.44; Figure 3d; Figures S11,S12; slower kinetics were also observed in single transfection experiments, Figure S16, Supporting Information). Given the interaction of TRD with MCAF/SETDB1 and other heterochromatin factors, this suggests that this domain facilitates hMBD1 association to heterochromatic loci as found in pericentromeric chromocenters.

**Figure 4:**
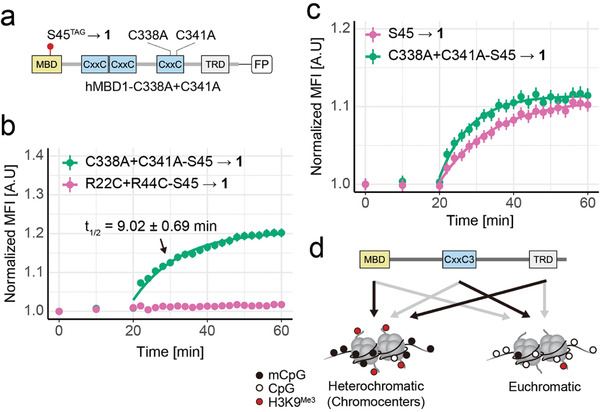
Studying the role of the hMBD1 CxxC3 domain in chromocenter association by light activation. a) Domain structure of hMBD1‐C338A+C341A‐ S45→**1** mutant. b) Time courses and fitting curves of chromocenter association after simultaneous light activation of hMBD1‐C338A+C341A‐S45→**1** and hMBD1‐R22C‐R44C‐S45→**1** co‐expressed in NIH/3T3 cells. (N = 509 chromocenters, 24 cells; *t*_1/2_ from three biological replicates totalling 1221 chromocenters of 52 cells). c) Time courses and fitting curves of experiments conducted as in Figure 4b with mCherry‐tagged hMBD1‐S45→**1** and EGFP‐tagged hMBD1‐C338A+C341A‐S45 →**1** co‐expressed in NIH/3T3 cells. (N = 702 chromocenters, 28 cells; *t*_1/2_ from three biological replicates totalling 961 chromocenters of 39 cells). d) Scheme of the proposed hypothesis for the role of the studied CxxC3 and TRD domains in hMBD1 chromocenter association.

hMBD1 exists in several splice isoforms featuring either two or three CxxC zinc‐finger domains.^[^ ^2^ ^]^ The third (CxxC3) selectively interacts with nonmethylated CpGs, equipping full‐length hMBD1 with two epigenetically opposed reader functions, and the ability to suppress both methylated and non‐methylated promoters.^[^ ^6^ ^]^ To gain insights into how the opposed CpG/5mCpG affinities of these two domains collectively regulate the association of hMBD1 to heterochromatic regions, we removed the CpG affinity of CxxC3 by introducing C338A and C341A mutations, which abolish its ability to coordinate Zn(II) and hence fold into a functional domain^[^ ^16^ ^]^ (**Figure** **4**a). We co‐expressed EGFP‐tagged hMBD1‐C338A+C341A‐S45→**1** with the mCherry‐tagged hMBD1‐R22C+R44C‐S45→**1** control and light‐decaged both as before. In kinetic studies conducted as above, the CxxC3 mutant exhibited slightly increased chromocenter association kinetics compared to wt. hMBD1 (*t*_1/2_ = 9.02 ± 0.69 min compared to *t*_1/2_ = 10.46 ± 0.81 min; Figures 4b and 3b; see also Figures S13,S14, Supporting Information for additional data). Since this difference in association kinetics was comparably small, we conducted additional experiments to quantify the kinetics of both hMBD1 mutants alone, or under competing conditions. In single transfections, the hMBD1‐C338A+C341A mutant alone consistently showed more rapid chromocenter association than wt. hMBD1 (Figure S16, Supporting Information, *t*_1/2_ = 6.26 ± 1.22 min compared to *t*_1/2_ = 10.75 ± 1.35 min).

In addition, the hMBD1‐C338A+C341A‐S45→**1** mutant also exhibited faster chromocenter association kinetics when co‐expressed and simultaneously light‐activated with wt. hMBD1‐S45→**1** (Figure 4c, *t*_1/2_ = 7.40 ± 1.37 min compared to *t*_1/2_ = 10.54 ± 1.80 min). Finally, we observed the same trend in an analogous experiment with switched fluorophores (Figure S15, Supporting Information, *t*_1/2_ = 7.58 ± 0.74 min compared to *t*_1/2_ = 11.84 ± 2.20 min). This increased chromocenter association kinetics of hMBD1 in absence of its nonmethylated CpG affinity implies a role of CxxC3 in controlling the distribution between CpG‐ and 5mCpG‐rich (i.e., eu‐ versus heterochromatic regions, Figure 4d). In consequence, this may control the response times between de novo or maintenance methylation events, and their downstream repressive output at 5mCpG‐rich heterochromatic regions such as chromocenters. The existence of natural hMBD1 splice variants differing in the presence of exactly this domain suggests an evolved regulatory function of these two alternative pathways.

## 3 Conclusion

In summary, we report the first approach to conditionally control the DNA affinity of an MBD, the central reader of mammalian DNA methylation. We identify a natural serine site conserved among all core family MBDs that is located at the DNA interface. Genetic encoding of **1** at this site enables the expression of transiently non‐mCpG binding hMBD1, and its scarless activation with a short light pulse, enabling measurements of rapid chromocenter association kinetics in mouse fibroblasts. By studying this association dependent on the function/presence of the regulatory CxxC3 and TRD domains of hMBD1, we reveal a kinetic modulation by both of these domains, arguing for a model in that the CxxC3 delays methylation responses of MBD1 by holding it at unmethylated loci outside of chromocenters, whereas the TRD promotes responses by aiding heterochromatin association. Our approach thus offers otherwise inaccessible kinetic insights into the complex, multidomain regulation of 5mC‐reading by a central MBD reader. The ability to control this initial step of the translation of 5mC into altered regulatory outputs with high temporal resolution sets the basis to further unravel the contribution of individual binding partners within the complex hMBD1 interaction network to the dynamics of methylation‐dependent rearrangements of mammalian chromatin. This includes other chromatin factors with DNA affinity or interactions to other DNA binders (e.g., TET1, SUV39H, HDAC1, etc.).

## Conflict of Interest

The authors declare no conflict of interest.

## Supporting information

Supporting Information

Supplemental Movie

## Data Availability

The data that support the findings of this study are available from the corresponding author upon reasonable request.
