# Supplementary material for: Light‐Activatable MBD‐Readers of 5‐Methylcytosine Reveal Domain‐Dependent Chromatin Association Kinetics In Vivo
Source: Adv Sci (Weinh). 2024 Jan 2;11(11):2307930. doi: 10.1002/advs.202307930 (PMC10953577; doi:10.1002/advs.202307930)
Supplement: Supplementary file 1 — Supporting Information [file ADVS-11-2307930-s002.pdf]

ADVANCED SCIENCE
Open Access

Supporting Information

for *Adv. Sci.*, DOI 10.1002/advs.202307930

Light-Activatable MBD-Readers of 5-Methylcytosine Reveal Domain-Dependent Chromatin Association Kinetics In Vivo

*Tzu-Chen Lin, Lena Engelhard, Benedikt Söldner, Rasmus Linser and Daniel Summerer**

Light-Activatable MBD-Readers of 5-Methylcytosine Reveal Domain-Dependent Chromatin Association Kinetics in Vivo

Tzu-Chen Lin[a], Lena Engelhard[a], Benedikt Sölder[a], Rasmus Linser[a] and Daniel Summerer*[a]

[a] Dr. T. Lin, B. Sölder, L. Engelhard, Prof. Dr. R. Linser, Prof. Dr. Daniel Summerer

Department of Chemistry and Chemical Biology, TU Dortmund University
Otto-Hahn Str. 4a, 44227 Dortmund
E-mail: daniel.summerer@tu-dortmund.de

Supplementary Information

TABLE OF CONTENTS

SUPPLEMENTARY FIGURES

- Figure S1. Representative FCM density plots showing the incorporation of 1 for indicated hMBD1 constructs and sorting strategies. 4
- Figure S2. Co-localization of hMBD1 wt and T27S mutant in NIH/3T3 cells. 5
- Figure S3. Decaging efficiency of 1 (0.5 mM in DPBS). 6
- Figure S4. Titrating the light dose for activating caged hMBD1 in live NIH/3T3 cells. 7
- Figure S5. In vitro binding affinity of caged and uncaged hMBD1 to methylated and non-methylated DNA. 8
- Figure S6. Light activation of EGFP-tagged hMBD1-S45→1 in live NIH/3T3 cells. 9
- Figure S7. Simultaneous light activation of hMBD1 S45→1 and T27→1 in live NIH/3T3 cells. 10
- Figure S8. Foci number quantification of simultaneous light activation of EGFP-tagged hMBD1-S45→1 and mCherry-tagged hMBD1-R22C+R44C-S45→1 in live NIH/3T3 cell. 11
- Figure S9. Supplementary cell images and kinetic analysis of simultaneous light activation of EGFP-tagged hMBD1-S45→1 and mCherry-tagged hMBD1-R22C+R44C-S45→1 in live NIH/3T3 cells. 12
- Figure S10. Simultaneous light activation of hMBD1-S45→1 and hMBD1-R22C+R44C-S45→1 with switched C-terminal fluorophore in live NIH/3T3 cells. 13
- Figure S11. Supplementary cell images and kinetic analysis of simultaneous light activation of EGFP-tagged hMBD1-ΔTRD-S45→1 and mCherry-tagged hMBD1-R22C+R44C-S45→1 in live NIH/3T3 cells. 14
- Figure S12. Simultaneous light activation of hMBD1-ΔTRD-S45→1 and hMBD1-R22C+R44C-S45→1 with switched C-terminal fluorophore in live NIH/3T3 cells. 15
- Figure S13. Supplementary cell images and kinetic analysis of simultaneous light activation of EGFP-tagged hMBD1-C338A+C341A-S45→1 and mCherry-tagged hMBD1-R22C+R44C-S45→1 in live NIH/3T3 cells. 16
- Figure S14. Simultaneous light activation of hMBD1-C338A+C341A-S45→1 and hMBD1-R22C+R44C-S45→1 observed with switched C-terminal fluorophore in live NIH/3T3 cells. 17
- Figure S15. Simultaneous light activation of hMBD1-S45→1 and hMBD1-C338A+C341A-S45→1 in live NIH/3T3 cells. 18
- Figure S16. Kinetic analysis of the chromocenter association of the EGFP-tagged hMBD1 wt, ΔTRD mutant, and the CXXC3 mutant following light activation (single transfections). 20
- Figure S17. Schematic illustration of the image analysis workflow. 21
- Figure S18. Plasmid maps of the expression vectors for hMBD1-S45^{TAG}. 22

MATERIAL AND METHODS

- Vector Construction 23
- Cell culture and transfection 24

- Fluorescence-activated cell sorting (FACS) and light-activation of hMBD1 24
- FCM Data analysis with R 25
- Image quantification and kinetic curve fitting 25
- Molecular simulation and energy minimization of hMBD1-S45 and T27→1 26
- Protein-DNA interaction assay by affinity pulldown 26
- Decaging 1 in solution 27

SUPPLEMENTARY TABLES

- Table S1. Oligonucleotides for plasmids construction. 28
- Table S2. Protein coding sequences used in this study 28
- Table S3. Oligonucleotides for protein-DNA interaction assay. 30
- Table S4. Colocalization analysis with Pearson's correlation coefficient for Figure 2. 30
- Table S5. Foci number analysis for Figure 2. 30

SUPPLEMENTARY REFERENCES 26

SUPPLEMENTARY FIGURES

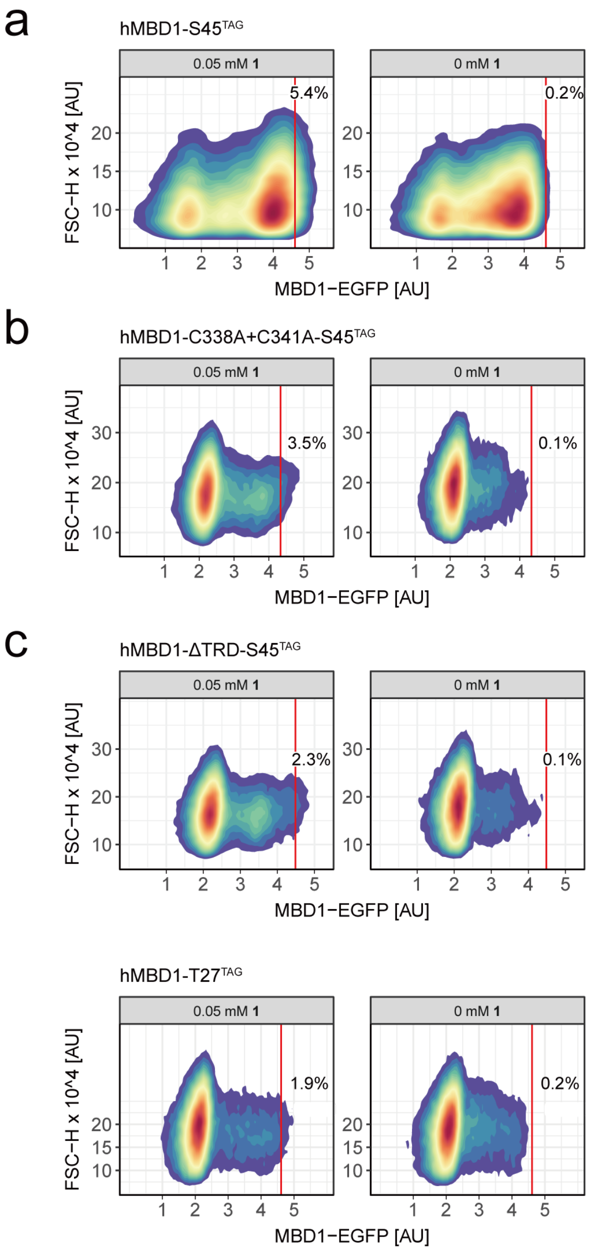

Figure S1. Representative FCM density plots showing the incorporation of 1 for indicated hMBD1 constructs and sorting strategies.

The EGFP fluorescence of HEK293T cells co-transfected with vectors encoding LRS/$tRNA^{Leu}$ and **a**) hMBD1-$S45^{TAG}$ **b**) hMBD1-C338A+C341A-$S45^{TAG}$ **c**) hMBD1-ΔTRD-$S45^{TAG}$, or **d**) hMBD1-$T27^{TAG}$ and grown in the presence (left) or absence (right) of 0.05 mM **1** were analyzed by FCM 24 h after transfection. Red lines indicate the intensity thresholds determined by the fluorescence intensity of cells grown in the absence of **1** (right). The determined thresholds were used for sorting cell populations that successfully incorporated **1**. Two independent biological replicates were performed.

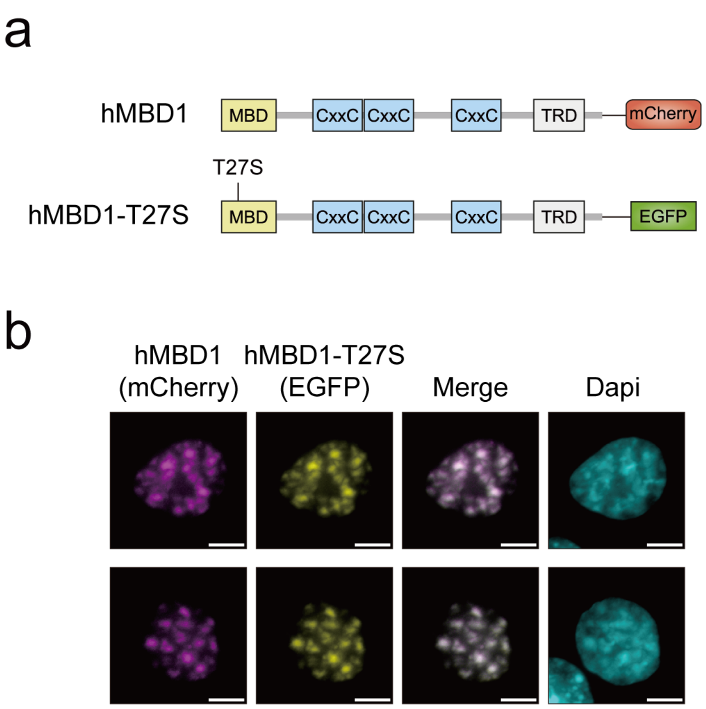

Figure S2. Co-localization of hMBD1 wt and T27S mutant in NIH/3T3 cells.

a) Domain structure of C-terminally mCherry-tagged hMBD1 wt and C-terminally EGFP-tagged hMBD1 T27S mutant. **b**) Co-expression of mCherry-tagged hMBD1 wt and EGFP-tagged hMBD1 T27S mutant in two exemplary NIH/3T3 cells. Merged images show their co-localization (magenta: hMBD1 wt, yellow: hMBD1 T27S) at Dapi-dense chromocenters in the nucleus, indicating that the T27S point mutation does not influence the 5mCpG recognition of hMBD1. Scale bar: 5 µm.

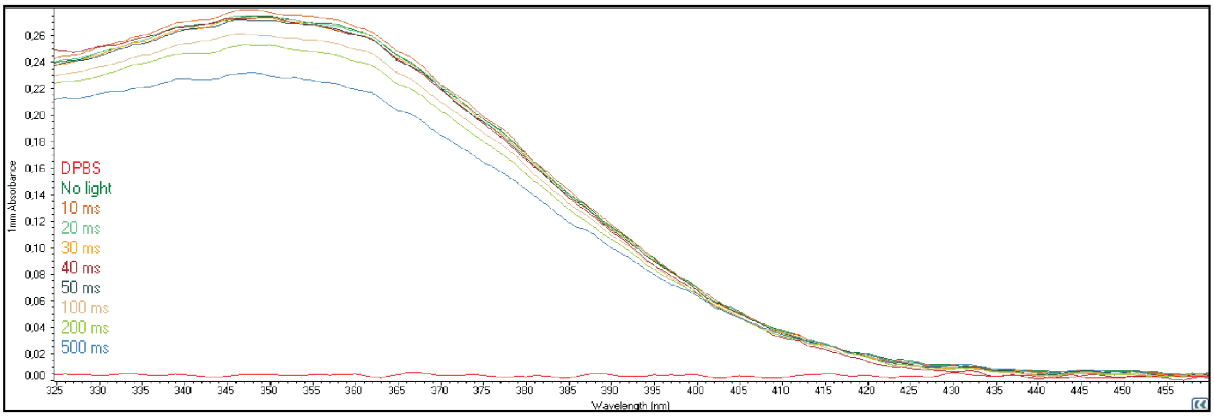

Figure S3. Decaging efficiency of 1 (0.5 mM in DPBS).

In vitro decaging experiment was conducted with the same light source used in all cell experiments. Different exposure time was applied to irradiate 100 μL sample in the flat-bottom cell culture plate (same as other cell experiments) to obtain the decaging efficiency.

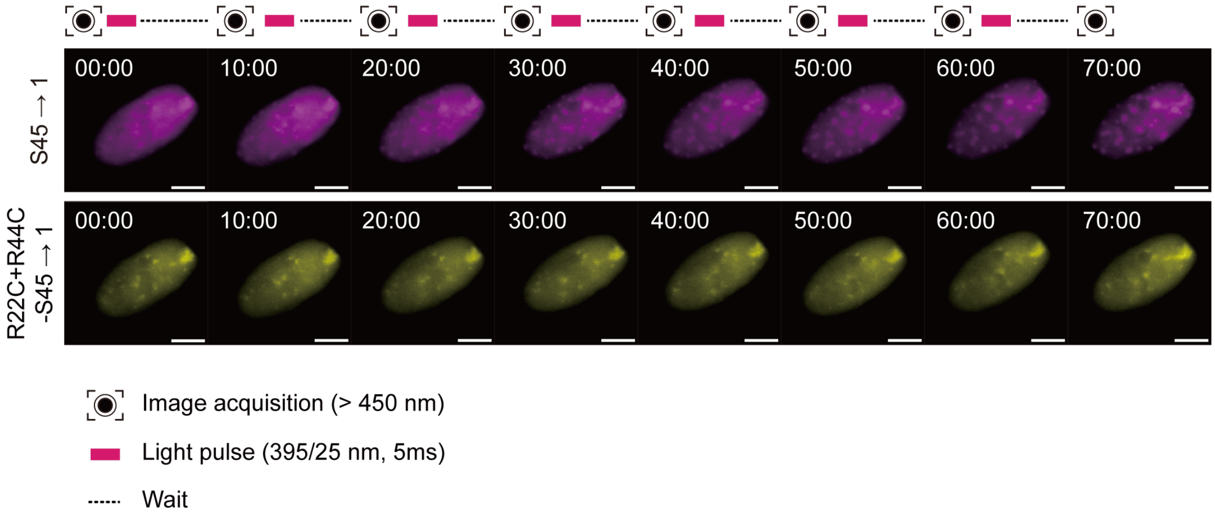

Figure S4. Titrating the light dose for activating caged hMBD1 in live NIH/3T3 cells.

NIH/3T3 cells co-expressing hMBD1-S45→**1** and hMBD1-R22C+R44C-S45→**1** were repeatedly irradiated for 5 ms with light (395/25 nm, 100% intensity, LED) in a 10 min interval, and images were acquired at 10 minutes after each light pulse to allow sufficient time for the chromocenter binding of uncaged hMBD1. Fluorescence saturation at the chromocenters was observed after 50 minutes, indicating that at duration of at least 30 ms of a full intensity (100%) light pulse is necessary to fully uncage hMBD1-S45→**1**. Notably, significant photobleaching or decreased cell viability due to phototoxicity were not observed after a total of 50 ms full intensity light pulse. Scale bar: 5 µm.

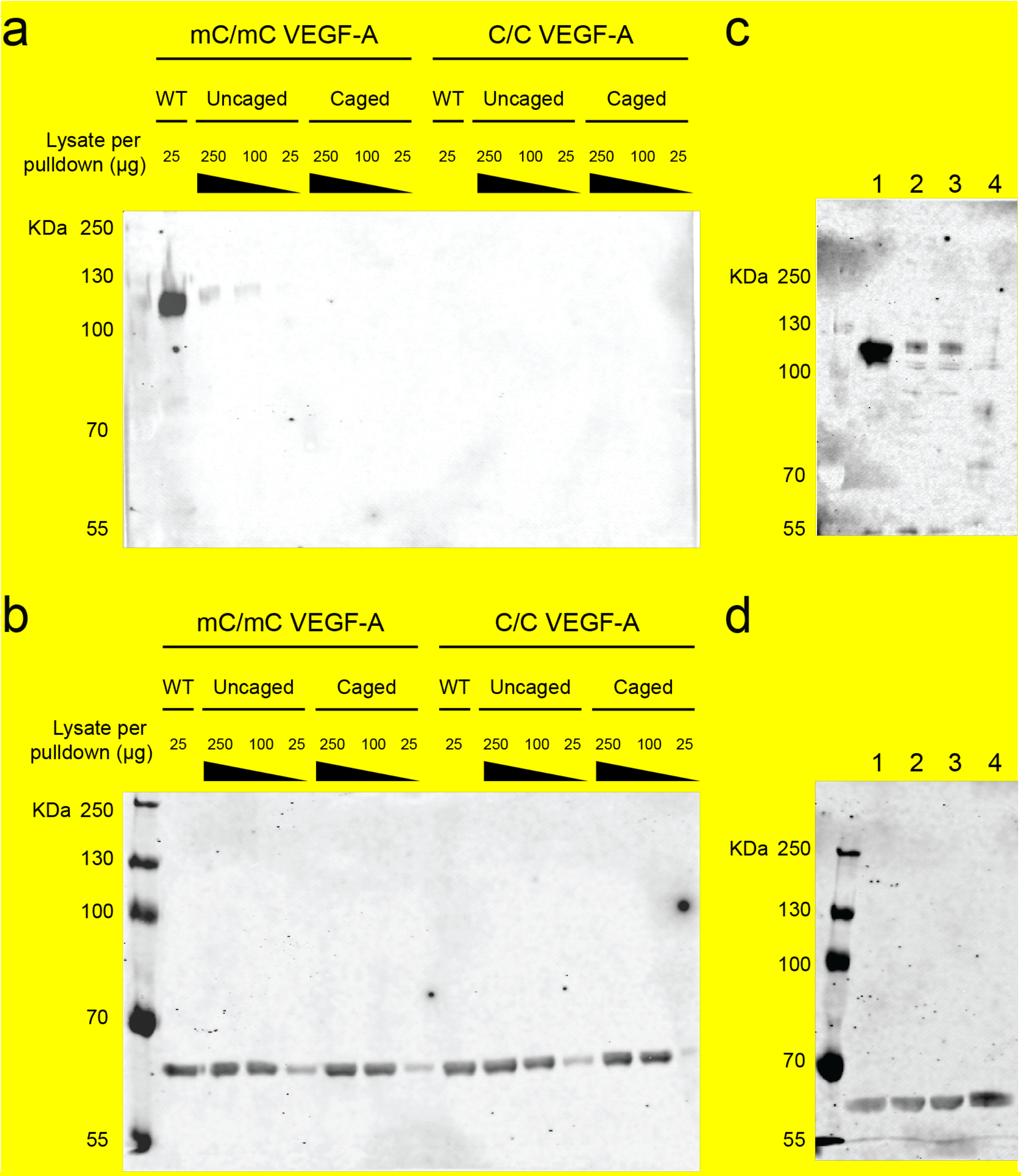

Figure S5. In vitro binding affinity of caged and uncaged hMBD1 to methylated and non-methylated DNA.

a) The overexpressed hMBD1 and S45 → **1** mutant (either irradiated or not irradiated) in HEK293T cell lysates were use in pull-down assays with methylated and non-methylated VEGF-A probe and blotted for the C-terminal Myc-tag (hMBD1-EGFP-Myc construct: 97 KDa). b) Beta-tubulin in the pulldown supernatant was blotted as the loading control. c) The following HEK293T cell lysates were blotted with c) anti-Myc antibody and d) anti-beta-tubulin antibody as input control. 1: HEK293T cells expressing hMBD1; 2: HEK293T cells expressing hMBD1 S45 → **1** and irradiated with light; 3: HEK293T cells expressing hMBD1 S45 → **1**; 4: Non-transfected HEK293T cells. The experiment was independently repeated three times.

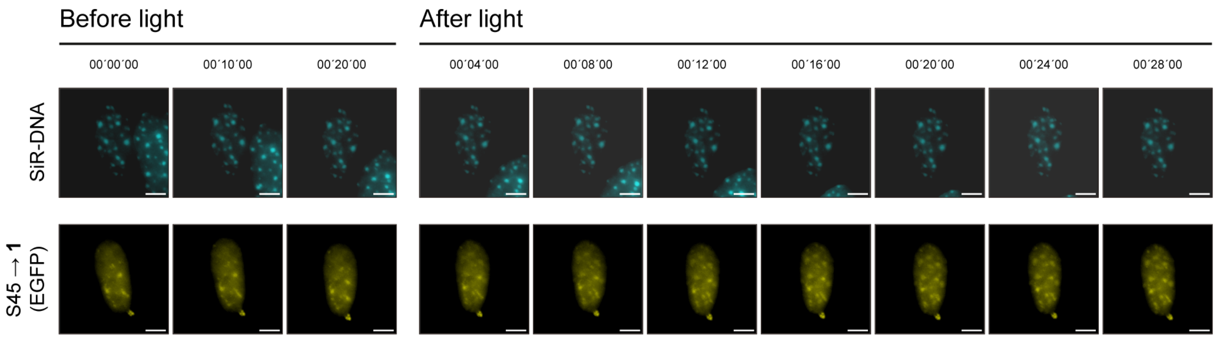

Figure S6. Light activation of EGFP-tagged hMBD1-S45→1 in live NIH/3T3 cells.

NIH/3T3 cells expressing EGFP-tagged hMBD1-S45→**1** were imaged for 20 minutes prior to uncaging. Subsequently, uncaging was performed with a light pulse (395/25 nm, 50 ms, LED), and the chromocenter association kinetics of hMBD1 was monitored over time in 4 min intervals. 5mCpG-rich chromocenters are indicated by foci in SiR-DNA nucleus stain. Scale bar: 5 µm.

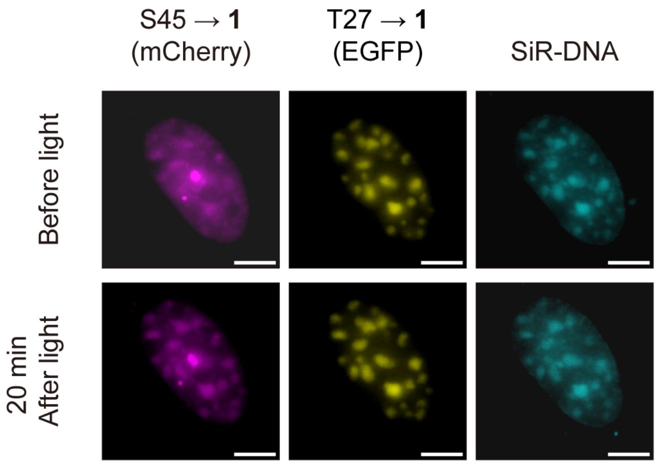

Figure S7. Simultaneous light activation of hMBD1 S45→1 and T27→1 in live NIH/3T3 cells.
Co-expression and simultaneous light activation of mCherry-tagged hMBD1-S45→**1** and EGFP-tagged hMBD1-T27→**1** in NIH/3T3 cells. 5mCpG-rich chromocenters are indicated by foci in SiR-DNA nucleus stain. While hMBD1-S45 did localized to chromocenters only after light activation, hMBD1-T27→**1** exhibited chromocenter localization already before light activation and remained unchanged after activation. Scale bar: 5 µm.

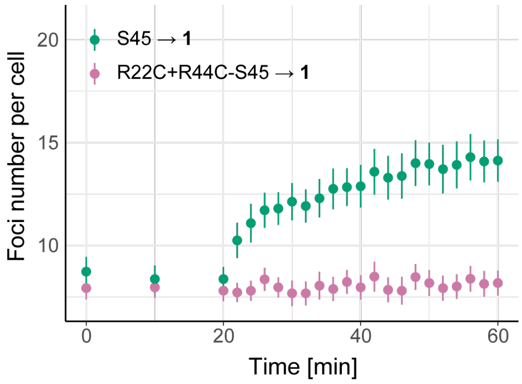

Figure S8. Foci number quantification of simultaneous light activation of EGFP-tagged hMBD1-S45→1 and mCherry-tagged hMBD1-R22C+R44C-S45→1 in live NIH/3T3 cell.

Time-resolved quantification of foci number per cell in NIH/3T3 cells co-expressing EGFP-tagged hMBD1-S45→**1** and mCherry-tagged hMBD1-R22C+R44C-S45→**1** before and after light activation. The mean and standard error of all cells from three independent experiments (N = 25 cells) are plotted.

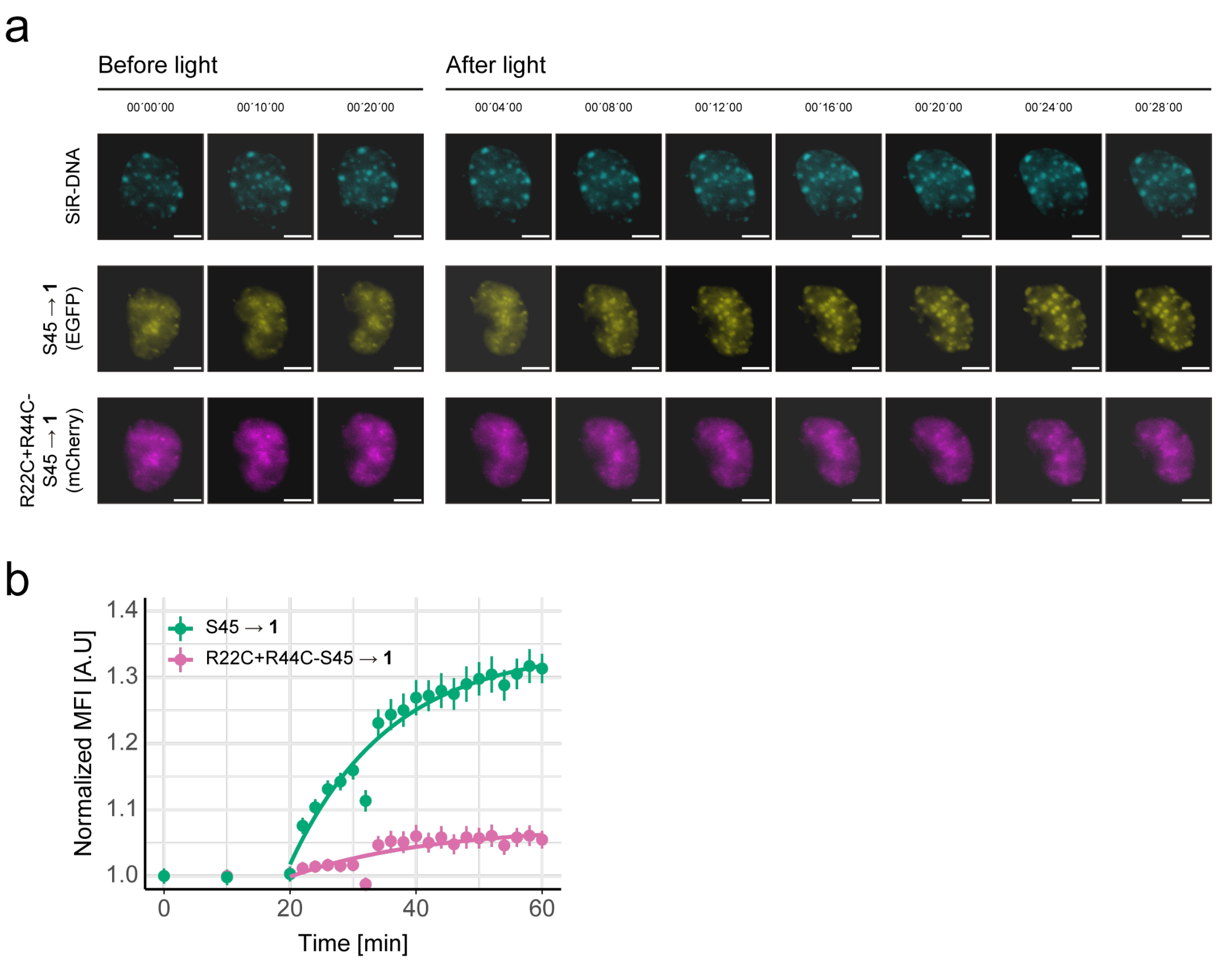

Figure S9. Supplementary cell images and kinetic analysis of simultaneous light activation of EGFP-tagged hMBD1-S45 → 1 and mCherry-tagged hMBD1-R22C+R44C-S45 → 1 in live NIH/3T3 cells.

a) Time-resolved imaging of cells co-expressing EGFP-tagged hMBD1-S45→**1** and mCherry-tagged hMBD1-R22C+R44C-S45→**1** before and after their simultaneous light activation (time in minutes). 5mCpG-rich chromocenters are indicated by foci in SiR-DNA nucleus stain. Scale bar: 5 µm. b) An independent replication of the time-resolved measurements of chromocenter fluorescence in NIH/3T3 cells co-expressing EGFP-tagged hMBD1-S45→**1** and mCherry-tagged hMBD1-R22C+R44C-S45→**1** before and after light activation. The mean, standard error, and the pseudo-first-order kinetic fitting curve of all normalized MFI values in chromocenters (N = 274 chromocenters, 13 cells) are plotted.

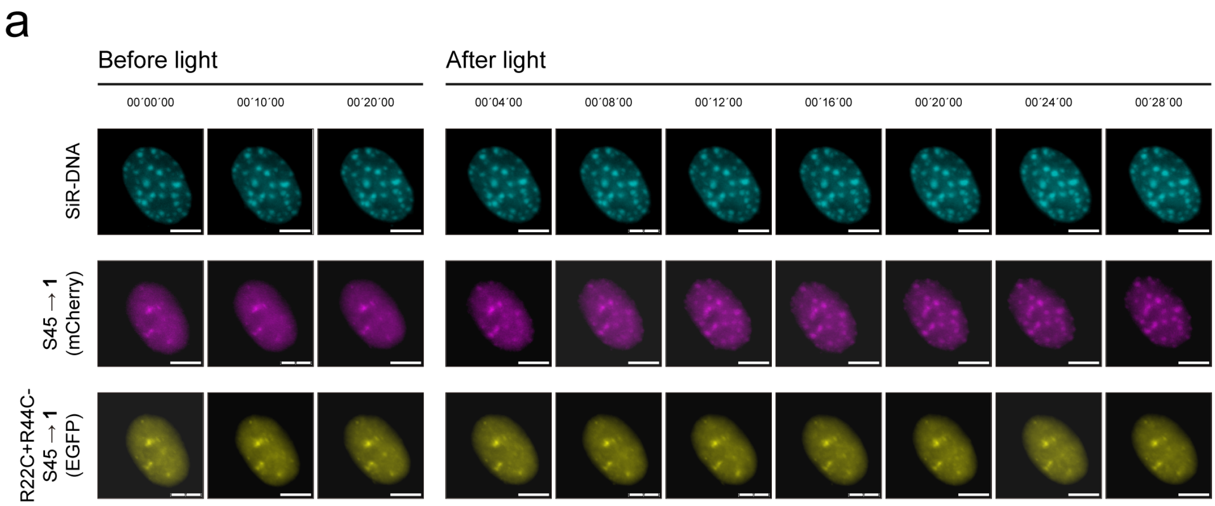

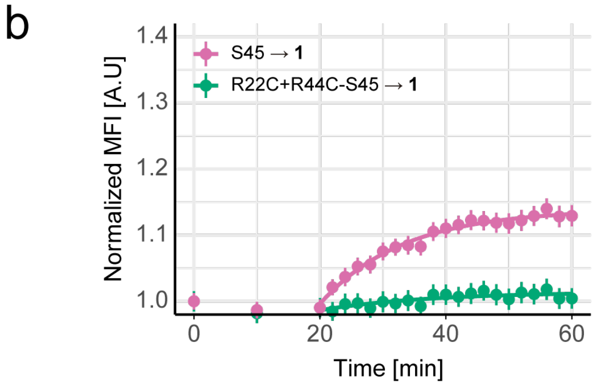

Figure S10. Simultaneous light activation of hMBD1-S45→1 and hMBD1-R22C+R44C-S45→1 with switched C-terminal fluorophore in live NIH/3T3 cells.

a) Time-resolved imaging of cells co-expressing mCherry-tagged hMBD1-S45→**1** and EGFP-tagged hMBD1-R22C+R44C-S45→**1** before and after their simultaneous light activation (time in minutes). 5mCpG-rich chromocenters are indicated by foci in SiR-DNA nucleus stain. Scale bar: 5 µm. b) Time-resolved measurements of chromocenter fluorescence in NIH/3T3 cells co-expressing mCherry-tagged hMBD1-S45→**1** and EGFP-tagged hMBD1-R22C+R44C-S45→**1** before and after light activation. The mean, standard error, and the pseudo-first-order kinetic fitting curve of all normalized MFI values in chromocenters (N = 191 chromocenters, 8 cells) are plotted. The experiment was replicated independently 3 times.

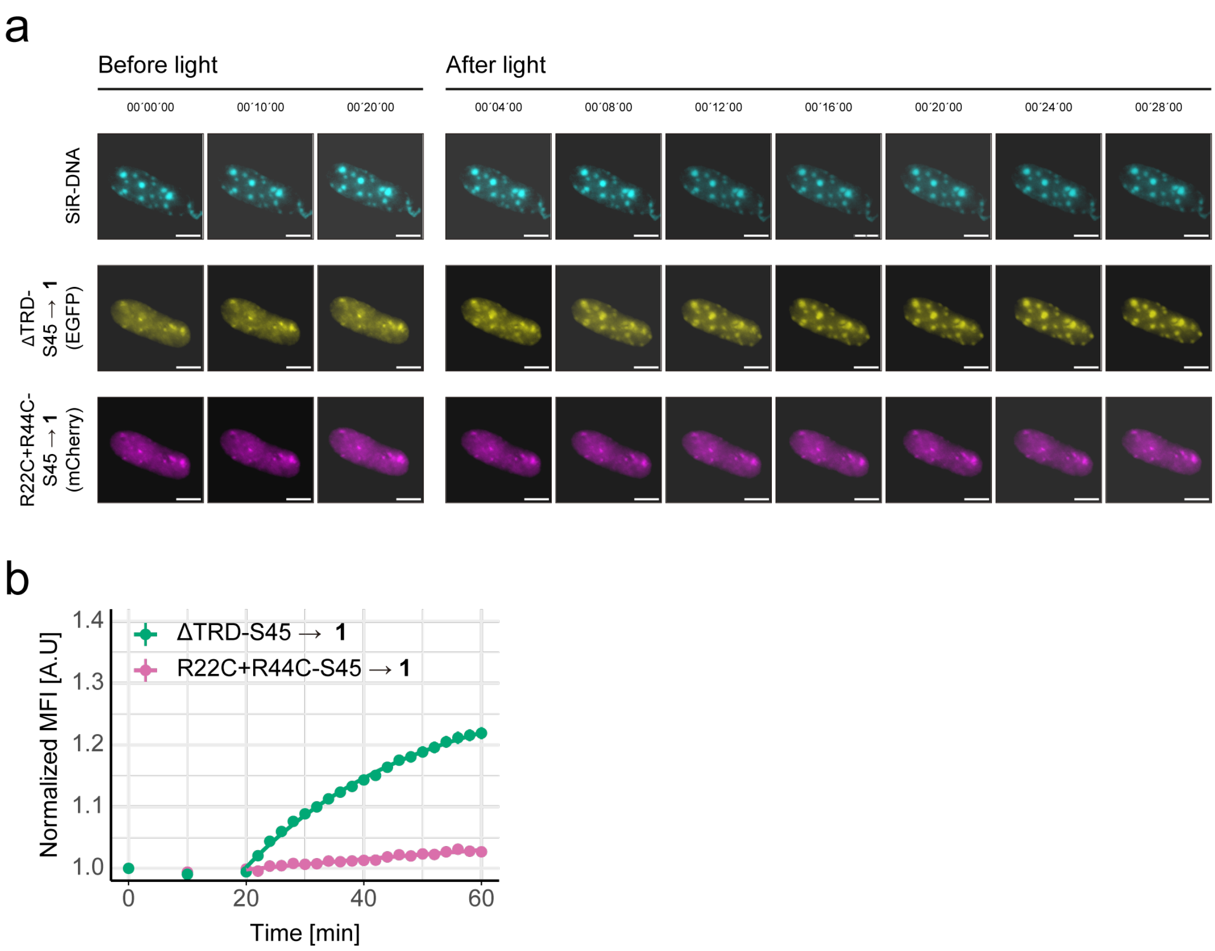

Figure S11. Supplementary cell images and kinetic analysis of simultaneous light activation of EGFP-tagged hMBD1-ΔTRD-S45→1 and mCherry-tagged hMBD1-R22C+R44C-S45→1 in live NIH/3T3 cells.

a) Time-resolved imaging of cells co-expressing EGFP-tagged hMBD1-ΔTRD-S45→**1** and mCherry-tagged hMBD1-R22C+R44C-S45→**1** before and after their simultaneous light activation (time in minutes). 5mCpG-rich chromocenters are indicated by foci in SiR-DNA nucleus stain. Scale bar: 5 µm. b) An independent replication of the time-resolved measurements of chromocenter fluorescence in NIH/3T3 cells co-expressing EGFP-tagged hMBD1-ΔTRD-S45→**1** and mCherry-tagged hMBD1-R22C+R44C-S45→**1** before and after light activation. The mean, standard error, and the pseudo-first-order kinetic fitting curve of all normalized MFI values in chromocenters (N = 503 chromocenters, 21 cells) are plotted.

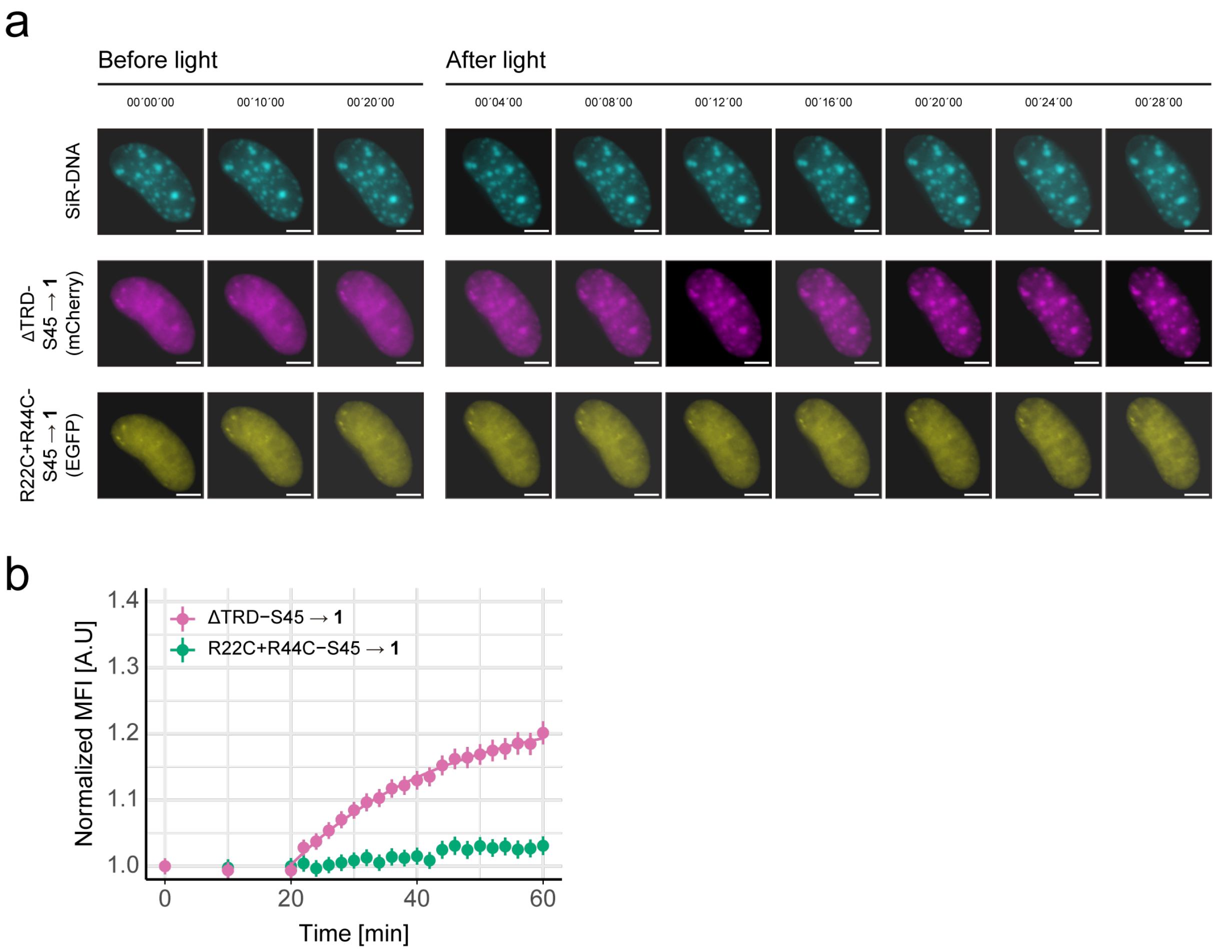

Figure S12. Simultaneous light activation of hMBD1-ΔTRD-S45→1 and hMBD1-R22C+R44C-S45→1 with switched C-terminal fluorophore in live NIH/3T3 cells.

a) Time-resolved imaging of cells co-expressing mCherry-tagged hMBD1-ΔTRD-S45→**1** and EGFP-tagged hMBD1-R22C+R44C-S45→**1** before and after their simultaneous light activation (time in minutes). 5mCpG-rich chromocenters are indicated by foci in SiR-DNA nucleus stain. Scale bar: 5 µm. b) Time-resolved measurements of chromocenter fluorescence in NIH/3T3 cells co-expressing mCherry-tagged hMBD1-ΔTRD-S45→**1** and EGFP-tagged hMBD1-R22C+R44C-S45→**1** before and after light activation. The mean, standard error, and the pseudo-first-order kinetic fitting curve of all normalized MFI values in chromocenters (N = 211 chromocenters, 10 cells) are plotted. The experiment was replicated independently 3 times.

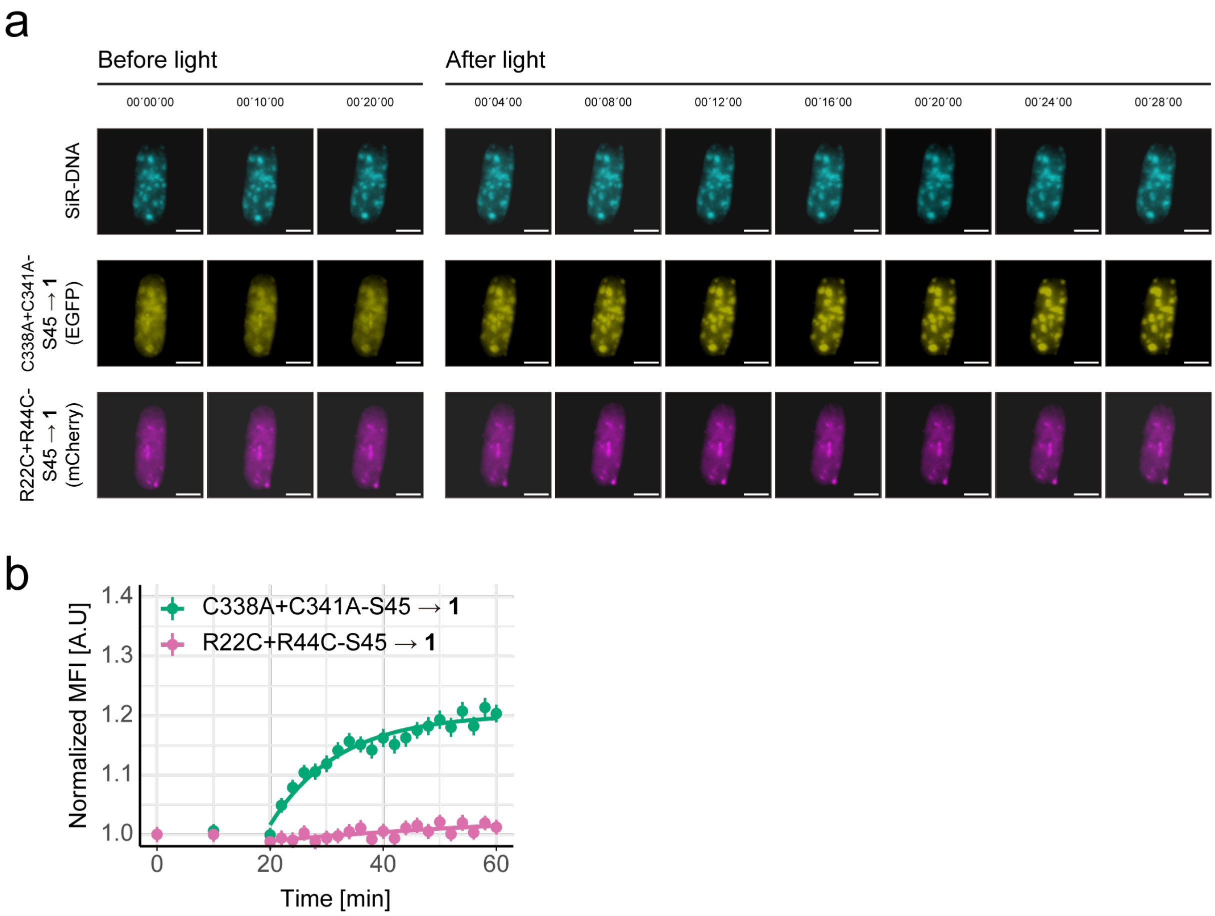

Figure S13. Supplementary cell images and kinetic analysis of simultaneous light activation of EGFP-tagged hMBD1-C338A+C341A-S45→1 and mCherry-tagged hMBD1-R22C+R44C-S45→1 in live NIH/3T3 cells.

a) Time-resolved imaging of cells co-expressing EGFP-tagged hMBD1-C338A+C341A-S45→**1** and mCherry-tagged hMBD1-R22C+R44C-S45→**1** before and after their simultaneous light activation (time in minutes). 5mCpG-rich chromocenters are indicated by foci SiR-DNA nucleus stain. Scale bar: 5 µm. b) An independent replication of the time-resolved measurements of chromocenter fluorescence in NIH/3T3 cells co-expressing EGFP-tagged hMBD1-C338A+C341A-S45→**1** and mCherry-tagged hMBD1-R22C+R44C-S45→**1** before and after light activation. The mean, standard error, and the pseudo-first-order kinetic fitting curve of all normalized MFI values in chromocenters (N = 234 chromocenters, 9 cells) are plotted.

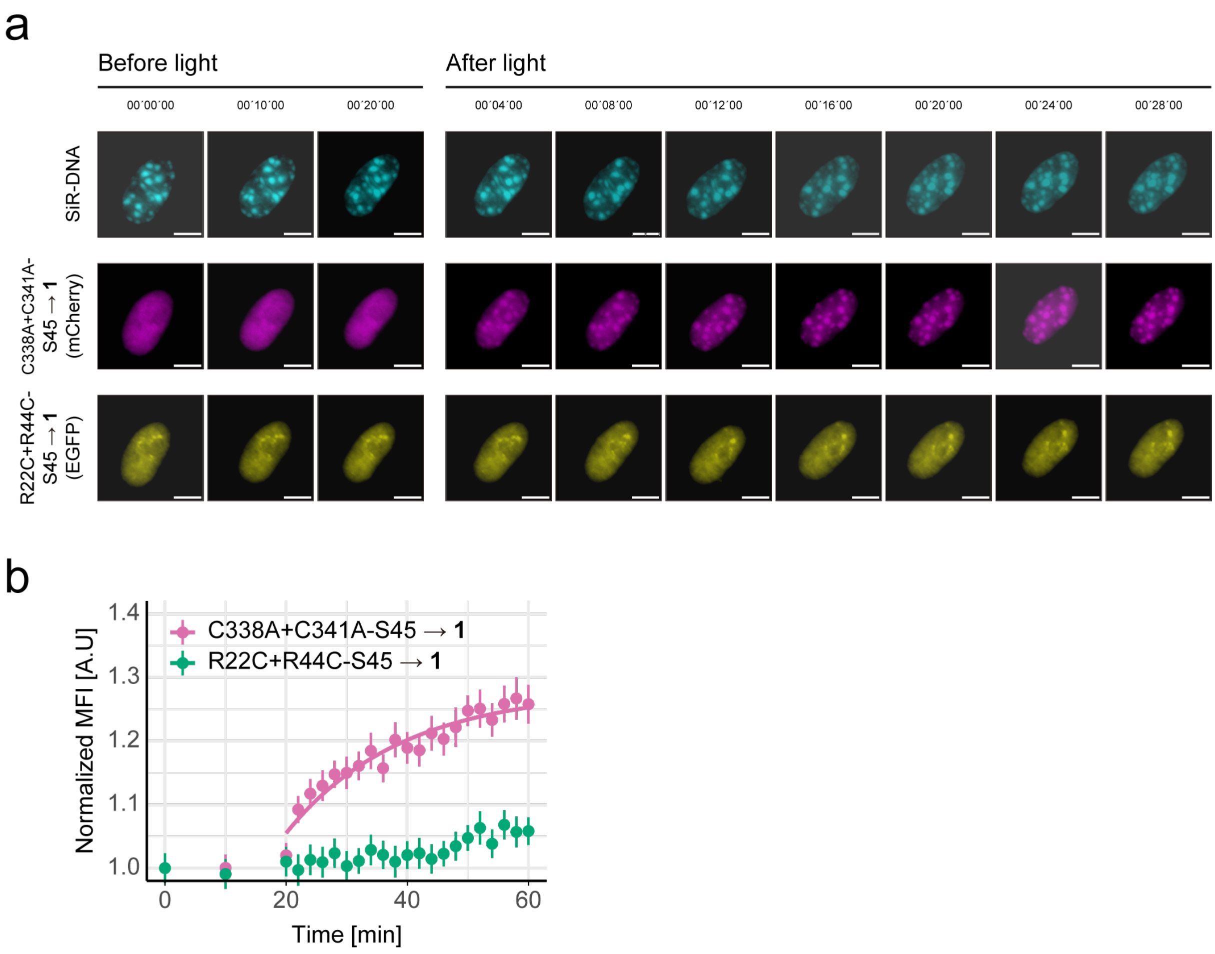

Figure S14. Simultaneous light activation of hMBD1-C338A+C341A-S45→1 and hMBD1-R22C+R44C-S45→1 observed with switched C-terminal fluorophore in live NIH/3T3 cells.

a) Time-resolved imaging of cells co-expressing mCherry-tagged hMBD1-C338A+C341A-S45→**1** and EGFP-tagged hMBD1-R22C+R44C-S45→**1** before and after their simultaneous light activation (time in minutes). 5mCpG-rich chromocenters are indicated by bright SiR-DNA nucleus stain. Scale bar: 5 µm. b) Time-resolved measurements of chromocenter fluorescence in NIH/3T3 cells co-expressing mCherry-tagged hMBD1-C338A+C341A-S45→**1** and EGFP-tagged hMBD1-R22C+R44C-S45→**1** before and after light activation. The mean, standard error, and the pseudo-first-order kinetic fitting curve of all normalized MFI values in chromocenters (N = 85 chromocenters, 3 cells) are plotted. The experiment was replicated independently 3 times.

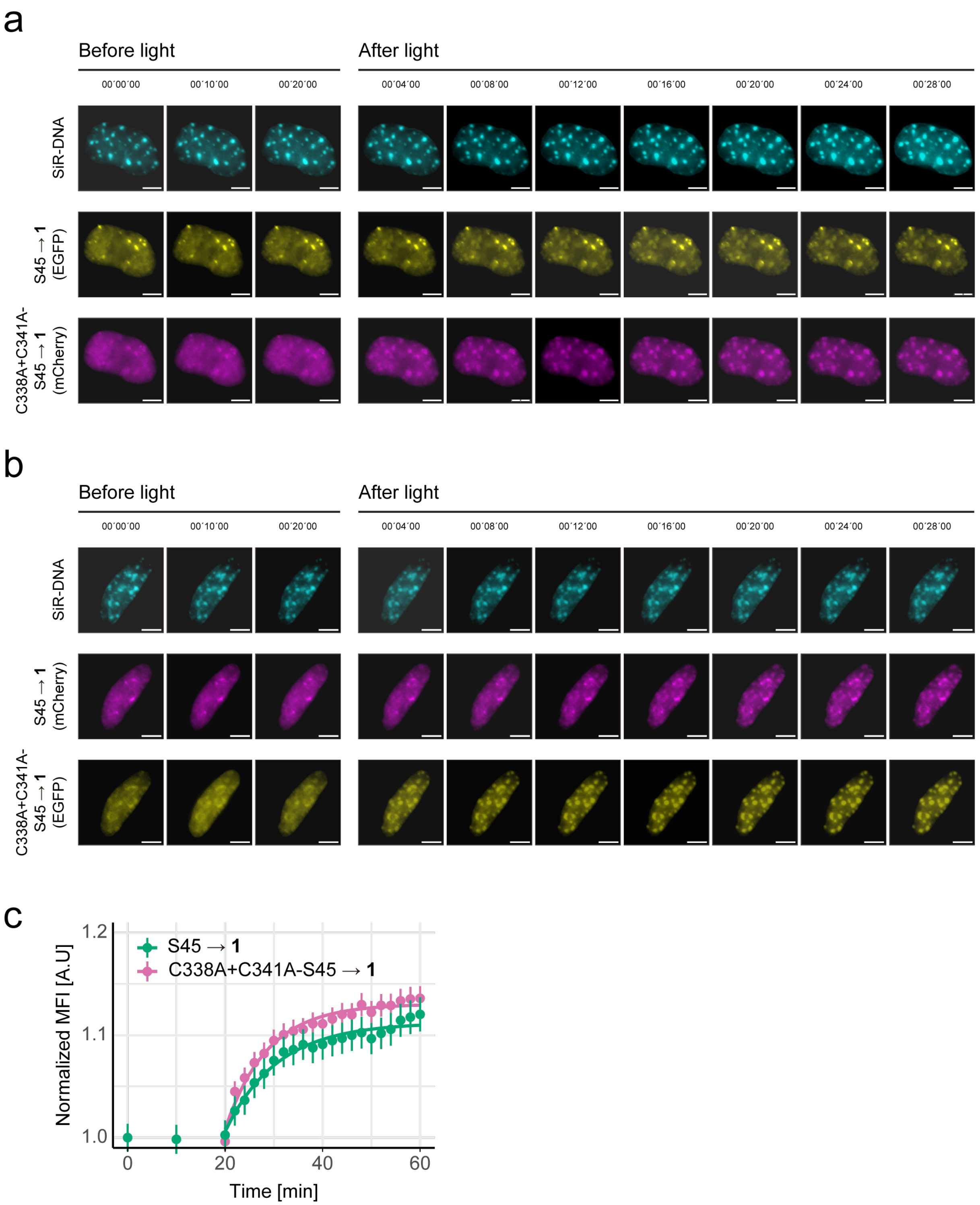

Figure S15. Simultaneous light activation of hMBD1-S45→1 and hMBD1-C338A+C341A-S45→1 in live NIH/3T3 cells.

Time-resolved imaging of cells co-expressing a) EGFP-tagged hMBD1-S45→**1** and mCherry-tagged hMBD1-C338A+C341A-S45→**1**, or b) mCherry-tagged hMBD1-S45→**1** and EGFP-tagged hMBD1-C338A+C341A-S45→**1** before and after their simultaneous light activation (time in minutes). 5mCpG-rich chromocenters are indicated by foci in SiR-DNA nucleus stain. Scale bar: 5 µm. c) Time-resolved measurements of chromocenter

fluorescence in NIH/3T3 cells co-expressing EGFP-tagged hMBD1-S45→**1** and mCherry-tagged hMBD1-C338A+C341A-S45→**1** before and after light activation. The mean, standard error, and the pseudo-first-order kinetic fitting curve of all normalized MFI values in chromocenters (N = 360 chromocenters, 13 cells) are plotted. The experiment was replicated independently 3 times.

a

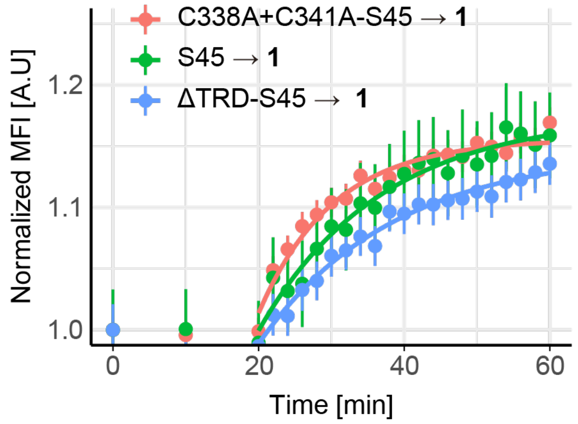

b

Construct	$t_{1/2}$ (min)
C338A+C341A-S45 → **1**	6.26 ± 1.22
S45 → **1**	10.75 ± 1.35
ΔTRD-S45 → **1**	13.17 ± 0.47

Figure S16. Kinetic analysis of the chromocenter association of the EGFP-tagged hMBD1 wt, ΔTRD mutant, and the CXXC3 mutant following light activation (single transfections).

a) Time-resolved measurements of chromocenter fluorescence in NIH/3T3 cells independently expressing either the EGFP-tagged hMBD1-S45→**1**, the EGFP-tagged hMBD1-ΔTRD-S45→**1**, or the EGFP-tagged hMBD1-C338A+C341A-S45→**1** before and after light activation. The mean, standard error, and the pseudo-first-order kinetic fitting curve of all normalized MFI values in chromocenters (for S45→**1**: N = 61 chromocenters, 3 cells; for ΔTRD-S45→**1**: N = 199 chromocenters, 9 cells; for C338A+C341A-S45→**1**: N = 413 chromocenters, 18 cells) are plotted. The experiment was replicated independently 2 times. b) The mean and standard deviation of $t_{1/2}$ (in minutes) of the EGFP-tagged hMBD1-S45→**1**, the EGFP-tagged hMBD1-ΔTRD-S45→**1**, and the EGFP-tagged hMBD1-C338A+C341A-S45→**1** were calculated from two biological replicates, summarizing 278 chromocenters from 11 cells, 308 chromocenters from 13 cells, and 475 chromocenters from 20 cells, respectively.

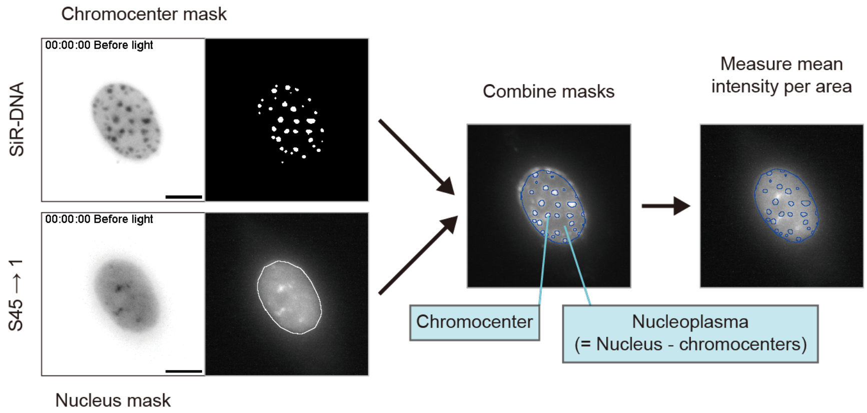

Figure S17. Schematic illustration of the image analysis workflow.

Firstly, chromocenter and nucleus masks were defined by images acquired from the SiR-DNA nucleus staining channel and the EGFP/mCherry fluorescence channel, respectively. The nucleus mask was used to confine the selection of chromocenters in order to exclude false positive chromocenter detection outside the nucleus. An additional "nucleoplasma" mask was created by subtracting the chromocenter areas from the nucleus area. The mean fluorescence intensities (MFI) of chromocenters or the nucleoplasma was measured from the images acquired by the EGFP and mCherry channels. Finally, the MFI of each chromocenter was normalized to the nucleoplasma MFI background of the corresponding nucleus to distinguish the chromocenter-specific and -non-specific localization. In kinetic measurements, these background-corrected MFI of each chromocenter were normalized to the average of normalized MFI value at t = 0, i.e. time point 20 min before irradiation.

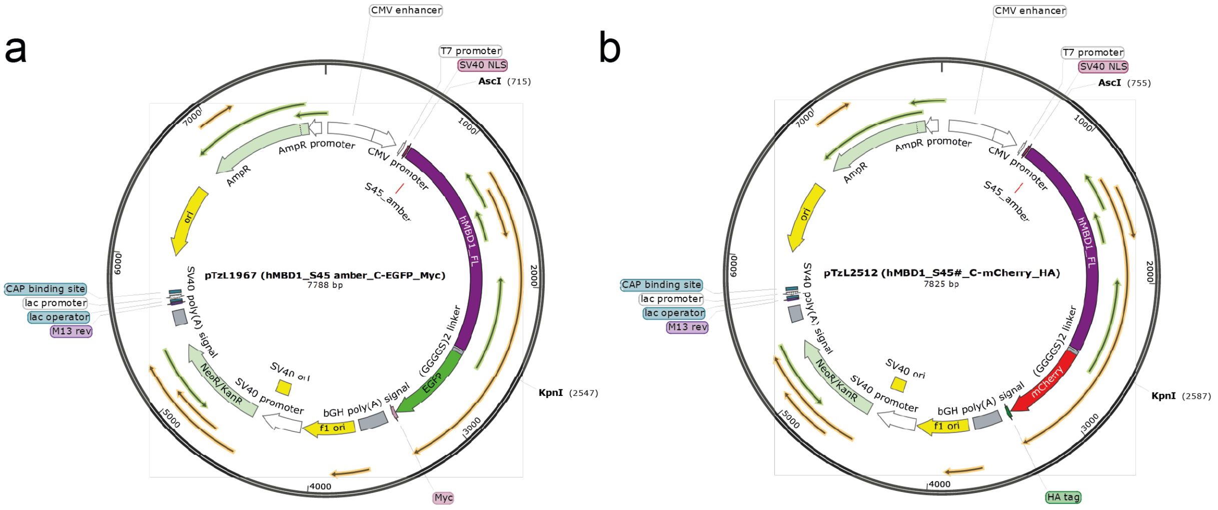

Figure S18. Plasmid maps of the expression vectors for hMBD1-S45^{TAG}.

Map of the expression vector for a) C-terminally EGFP-tagged hMBD1-S45^{TAG} b) C-terminally mCherry-tagged hMBD1-S45^{TAG}. The EGFP-tagged or mCherry-tagged domain mutants of hMBD1-S45^{TAG}, including hMBD1-R22C+R44C-S45^{TAG}, hMBD1-ΔTRD-S45^{TAG}, and hMBD1-C338A+C341A-S45^{TAG}, share the same vector features except for the indicated mutations.

MATERIALS AND METHODS

Vector Construction

All vectors were derived from pTzL1745 which is based on pcDNA3.1-GoldenGate-VP64 (Addgene 47389) with removed VP64 and lacZα gene as described previously [1,2]

To construct plasmids encoding EGFP-tagged hMBD1, a Myc tag was first introduced into pTzL1745 by quick change SDM using primers o3167/o3257, resulting in pTzL1746. The human full length MBD1 coding sequence was amplified from a human prostate cDNA library (BiocCt 10108-A-GVO-EB) using primers o3292/o3293, then MBD1 and EGFP (amplified with primers o3294/o3295) were assembled with pTzL1746 (amplified by primers o3290/o3291) via Gibson assembly, yielding pTzL1747. Finally, remaining unwanted sequences were either removed by quick change using primers o3642/o3643 to afford pTzL1836, or replaced with GGGGS linker by ligating o3387/o3388 using SacII/AscI to yield pTzL1833 (containing N-terminal Flag tags).

The C-terminally EGFP-tagged hMBD1 mutants were cloned as follows. The R22C mutation was introduced into pTzL1836 by quick change site-directed mutagenesis (SDM) using primers o3730/o3731 to yield pTzL1947. The R22C+R44C mutant was derived from pTzL1947 by introducing an R44C mutation with primers o3732/o3733 via quick change SDM to yield pTzL1964. The C338A+C341A mutations were introduced into pTzL1836 (hMBD1) using primers o4479/o4480, resulting in pTzL2645. The hMBD1-ΔTRD (aa 529-592 deleted) variant was cloned by Gibson assembly of 2 fragments amplified from pTzL1836 using primers o4302/o3291 and o3292/o4305, yielding pBiR2586. For EGFP-tagged amber mutant hMBD1-S45^{TAG}, the amber mutation was introduced into pTzL1836 by quick change SDM using primers o3756/o3757 to yield pTzL1967. The EGFP-tagged hMBD1-C338A+C341A-S45^{TAG} and hMBD1-ΔTRD-S45^{TAG} were cloned similarly by introducing amber mutation into pTzL2645 and pBiR2586 via quick change SDM using primers o3756/o3757 to yield pNaU2737 and pNaU2738, respectively. An additional hMBD1-S45^{TAG} plasmid including N-terminal Flag tags and GGGGS linker was cloned by restriction ligation of hMBD1-S45^{TAG} sequence (from pTzL1967) with vector backbone of pTzL1833 using AscI/KpnI, giving plasmid pTzL2511. The EGFP-tagged hMBD1-R22C+R44C-S45^{TAG} was cloned by introducing amber mutation into pTzL1964 via quick change SDM using primers o4128/o4129 giving pTzL2892. For the mCherry-tagged hMBD1, hMBD1-S45^{TAG}, hMBD1-C338A+C341A-S45^{TAG}, hMBD1-ΔTRD-S45^{TAG}, and hMBD1-R22C+R44C-S45^{TAG}, the coding sequences for hMBD1 and its mutants (from pTzL1836, pTzL1967, pNaU2737, pNaU2738, and pTzL2892, respectively) were ligated with the vector backbone of pTzL1960 [2] using AscI/KpnI, giving pTzL2512, pTzL2889, pTzL2890, and pTzL2902, respectively. The hMBD1-T27S mutant was cloned by introducing T27S mutation to pTzL1833 with primers o4594/o4560 via quick change SDM to afford pTzL2682. For EGFP-tagged amber mutant hMBD1-T27^{TAG}, the amber mutation was introduced into pTzL1833 by quick change SDM using primers o4592/o4558 to give pTzL2681.

The orthogonal *E. coli* leucyl synthetase (ecLRS-BH5) bearing the five previously reported mutations M40G, L41Q, Y499L, Y527G, H537F2 and the suppressor tRNA^{CUA} were encoded on the previously reported plasmid pStH1147 [1].

Cell culture and transfection

HEK293T cells were cultivated in DMEM (Dulbecco's Modified Eagle Medium, w/ 4.5 g/L Glucose, w/o: L-Glutamine, w: Sodium pyruvate, w: 3.7 g/L $NaHCO_3$, PAN Biotech, P04-03600) supplemented with 10% FBS (South America origin, premium grade, PAN Biotech, P30-3306), 2 mM L-glutamine (PAN Biotech, P04-80100), 100 U/mL of penicillin and 0.1 mg/mL of streptomycin (PAN Biotech, P06-07100) in a sterile humidified incubator (≥ 95%) at 37°C and a CO_2 level of 5%. For transfection, cells were seeded a day before to reach 70-80% confluency at the time of transfection. Transient plasmid transfection was carried out by the use of poly-ethyleneimine (PEI, 1mg/mL in dd H2O, pH 7) (linear MW 25.000, CAS 9002-98-6, Alfa Aesar).

Mouse embryonic fibroblast NIH/3T3 cells (ATCC, CRL-1658) were maintained in the same conditions described above. The plasmid transfection of NIH/3T3 was done either by PEI as described above, or by electroporation using the 100 μL Neon Transfection System (Invitrogen, Thermo Fisher Scientific Inc.). Briefly, 3,000,000 cells were resuspended in 100 μL resuspension buffer R (Neon-transfection 100 μL kit, Invitrogen, MPK10096) with 10–30 μg of plasmid and electroporated at a pulse voltage of 1350 volts, pulse width of 20 ms, and pulse number of 2. The cells were subsequently seeded in either 10 cm culture dishes or 96-well plates containing growth media (DMEM supplemented with 10% FBS and 2 mM L-glutamine as described above, w/o penicillin and streptomycin), then incubated in a humidified 37°C incubator with 5% CO_2.

Fluorescence-activated cell sorting (FACS) and light-activation of hMBD1

NIH/3T3 cells grown in 10 cm cell culture plate (Sarstedt) were transfected with plasmids encoding indicated MBD1-S45^{TAG} constructs (wt, R22C+R44C, C338A+C341A, or ΔTRD) and LeuRS/tRNA^{Leu} using either PEI or Neon-transfection system. For PEI transfection, growth media was exchanged with media supplemented with 0.05 mM **1** after 3 h of transfection and allowed further expression for 21 h. For Neon-transfection, cells were directly seeded in growth media containing 0.05 mM **1** after transfection and allowed expression for 24 h. Following transfection, cells successfully expressed caged hMBD1 (and its domain mutants) were distinguished and sorted by FACS. The cells were trypsinized from the culture dish, washed once with DPBS, pelleted by centrifugation, resuspended in 500 μL warm DPBS containing 1% BSA, and subjected to cell strainer before sorting. Cell sorting was performed with Sony Cell Sorter model LE-SH800SFP using 488 and 561 nm laser coupled with 525/50 and 617/30 nm filter to detect EGFP and mCherry, respectively. To afford the desired population expressing caged hMBD1, cells similarly transfected but grown in the absence of **1** were used as the negative control to determine fluorescence intensity thresholds in cell sorting. During cell sorting, cells were kept at 37ºC in the sample loading chamber before subjected to flow system (sample pressure 4, flow rate 21 μL/min). Sorted cell population was collected at RT into a 15 mL tube containing 6 mL warm (or RT) imaging media (DMEM containing 4.5 g/L glucose, stable glutamine, sodium pyruvate, 0.5g/L NaHCO3, and 25 mM HEPES from PAN Biotech, P04-01163, supplemented with 10% FBS). The collected cells were pelleted and resuspended in warm conditioned media, then approximately 500–2,000 cells per well were seeded into the black 96-well plate with flat polymer coverslip bottom (ibidi, 89626) pre-treated with 0.01% poly-L-lysine (CAS 25988-63-0, Sigma-Aldrich, P1274). Finally, cells were incubated in a humidified 37°C incubator with 5% CO_2 for 4-5 hours to allow adherence.

Live cell imaging was performed after cells have adhered and recovered from cell sorting. Before imaging experiments, DNA staining was performed by incubating cells with 1 μM SiR-DNA (SiR-DNA kit from Spirochrome AG, SC007) and 5 μM Verapamil (supplemented by the SiR-DNA kit) in growth media for 1 h in

the incubator (37°C, 5% CO2) prior to experiment. Cells were maintained in the same media (containing SiR-DNA and Verapamil) at 37ºC during live cell imaging.

Imaging and light-activation experiments were performed using an Olympus IX81 microscope equipped with a Hamamatsu model C10600-10B-H camera. Samples were illuminated with a Lumencor SPECTRA X light engine® NIR version which operates 6 independent LED light sources coupled with bandpass filters (V: 395/25 nm; B: 440/20 nm; C: 470/24; GY: 550/15 nm; R: 640/30 nm; TN: 730/40 nm). Images were acquired using a 60x oil immersion objective as z-stack images (0.5 µm/step) for EGFP (excitation with C, emission filter 554/23 nm), mCherry (excitation with GY, emission filter 635/18), DAPI (excitation with V, emission filter 474/27 nm), and SiR-DNA (excitation with R, emission filter nm).

Light activation of caged hMBD1 and its domain mutants were carried out with a user-defined automatic acquisition sequence in the cellSens imaging software (cellsens Dimension Version 3, Olympus). The automatic sequence started with image acquisition at 0, 10, and 20 min to monitor the nuclear localization of caged hMBD1 before light. Right after image acquisition at 20 min, light activation was performed by taking a snapshot using the excitation settings of DAPI channel (395/25 nm violet light illumination, 50 ms exposure with 100% intensity). Subsequently, post-activation cell images were acquired for a total of 40 min with 2 min interval.

FCM Data analysis with R

Flow cytometry standard files (FCS 3.0 or 3.1) were processed with R 4.1.2 in Rstudio (2022.02.3+492) using following Bioconductor packages: flowCore (2.0.0) [3], flowClust (3.26.0) [4,5], flowDensity (1.22.0) [6], flowStats (4.0.0) [7], and ggcyto (1.16.0) [8]. Fluorescence intensity data extracted from populations of interest were then analyzed using Tidyverse packages (1.3.1). In brief, cell populations were identified firstly from multivariate t mixture models, then singlet events were selected by a robust linear model with rlm. Populations showing positive fluorescence signals were distinguished by applying thresholds determined from respective negative controls (the upper boundary including 99.9% population in respective channels accordingly to the density distribution). Then the percentage of positive population in individual sample was calculated and plotted.

Image quantification and kinetic curve fitting

The microscopy image stacks were first Z-projected by maximal intensity (1344 x 1024 pixels, 32 bits), then the images were processed sequentially with background subtraction (convoluted background subtraction from BioVoxxel toolbox [9]), contrast enhance, smoothing (Gaussian blur), and thresholding using the Fiji distribution of ImageJ (1.53q) [10].

To perform the colocalization analysis, a nuclear mask was first selected in images of the overexpressed nuclear proteins acquired in green (EGFP) or red (mCherry) channels. Then a Fiji's plugin Coloc 2 was used to calculate the Pearson's correlation coefficient within the previously selected nuclear mask.

To analyze changes of the chromocenter fluorescence, the nuclear masks were selected in images of the overexpressed nuclear proteins acquired in green (EGFP) or red (mCherry) channels, then the chromocenters were identified in the DNA staining images (SiR-DNA, acquired in Cy5 channel) within each nuclear mask. An additional nucleoplasma mask was created by the XOR (exclusive or) selection of the nucleus mask and the chromocenter mask. Finally, mean fluorescence intensity (MFI) measurements were conducted for all selected region in green (EGFP) and red (mCherry) channels.

The kinetic analysis of obtained MFIs was performed with R 4.1.2 in Rstudio (2022.02.3+492) using Tidyverse packages (1.3.1). In brief, the chromocenter MFIs were first normalized to the nucleoplasma MFI of the corresponding nucleus, then the normalized MFI values of each time point in the time series were further normalized to the average of normalized MFI value at time = 0, giving normalized chromocenter MFIs. The resulting values were fitted using Equation 1 with the non-linear least square function *nls* to estimate chromocenter-binding half-lives ($t_{1/2}$).

$$y = y_0 + (y_{max} - y_0)(1 - e^{-kt})$$

Equation 1. Pseudo-first-order association kinetics. y = Normalized chromocenter MFI at time t; y_0 = Normalized chromocenter MFI at the initiation time point (time of activation, $t = 20$ min); y_{max} = Plateau MFI; k = Rate constant; $t_{1/2}$ = Half-life of saturation ($= (\ln 2)/k$).

Molecular simulation and energy minimization of hMBD1-S45 and T27→1

Models of hMBD1 mutated at positions T27 and S45 each with the ncAA **1** (4,5-dimethoxy-2-nitrobenzyl-L-serine) were energy minimized with Gromacs version 2020.1 [11]. Model 1 of the solution NMR structure of PDB 1IG4 [12] was used as template. For generating the mutation at position T27, threonine was at first replaced by serine using the rotamers tool with the Dunbrack 2010 backbone-dependent rotamer library [13] implemented in UCSF Chimera [14]. The rotamer with the highest predicted probability was chosen. In the second step, for both cases the mutation was introduced using the "Build Structure" tool implemented in UCSF Chimera. For reflecting the conformational flexibility of the mutated residues, for both mutated models, two additional, alternative starting structures for the energy minimization were generated each by varying the chi1 sidechain dihedral angle about +/- 120 degree.

The six starting structures were energy minimized with Gromacs version 2020.1 [11] using the latest Charmm36 [15,16] all-atom force field from February 2021. The parameters needed to implement the ncAA **1** into the force field were derived from CGenFF [16–19]. The protein models were solvated within a periodic dodecahedron simulation box using TIP4P water molecules. Steepest descent energy minimization was performed until the maximum force fall below 1000 kJ mol^{-1} nm^{-1}. Lennard-Jones 6-12 interactions were smoothly shifted to zero by starting to switch at 1.0 nm until a cutoff distance of 1.2 nm was reached. Coulomb interactions were treated with the Particle-Mesh Ewald (SPME) electrostatics using a cut-off for long-range electrostatics of 1.2 nm. The molecular graphics of model illustration were produced by UCSF ChimeraX [20,21].

Protein-DNA interaction assay by affinity pulldown

HEK293T cells grown in 10 cm cell culture plate (Sarstedt) were transfected with plasmids encoding C-terminally EGFP- and Myc-tagged wt hMBD1 or hMBD1-$S45^{TAG}$ using PEI. For the expression of wt hMBD1, the cells were incubated with the transfection mixture for 24 h before harvesting. For the expression of hMBD1-S45 → **1**, the LeuRS/$tRNA^{Leu}$ construct was co-transfected and cells were grown in the presence of 0.05 mM **1** for 24 h. Following transfection, cells expressing hMBD1-S45 → **1** were separated into two groups: one group was irradiated with 365 nm uv light (Witeg DH.WUV00010, 6x 15 W) for 5 min in warm DPBS to uncage the protecting group before harvesting, while the other group was harvested without uncaging. Followed by

trypsinization and centrifugation, the cell pellet was washed two times with ice-cold DPBS and incubated with ice-cold lysis buffer (2.5 mM Tris-HCl pH 8.0, 500 mM NaCl, 50 mM glucose, 10 mM EDTA, 0.2% NP40, 0.2% Tween 20) supplemented with protease inhibitor cocktail (cOmplete mini easy pack, Roche, 4693124001) for 10 min on ice. The lysed suspension was passed through a ∅0.8 needle for 5 times, followed by a ∅0.6 needle for 5 times and incubated for another 20 min on ice. Following centrifugation at 13,400 g for 20 min at 4 °C, the supernatant was snapped-frozen and stored at -80 °C before use.

The biotinylated DNA probes were generated by PCR using primers o4681 (5'-biotinylated)/o4666 to amplify the 201-bp long partial sequence of the human VEGF-A (vascular endothelial growth factor A) promoter sequence. The PCR reaction was performed with either the normal dNTP mix (NEB, N0447L) or the 5mC dNTP mix (Biozol, ZYM-D1030) to generate the non-methylated and methylated VEGF-A probes, respectively.

The VEGF-A probes were subsequently immobilized on the streptavidin-modified magnetic beads (Dynabeads™ MyOne™ Streptavidin T1, Invitrogen, 65601). 50 µg beads was washed and resuspended in 2x binding and washing buffer (10 mM Tris-HCl pH 7.5, 2 M NaCl, 1 mM EDTA). Following addition of equal volume DNA solution (containing 500 ng methylated or non-methylated VEGF-A), the beads were incubated for 1 h at room temperature with rotation. Then the beads were washed 3 times with 1x binding and washing buffer, followed by 2 times of protein binding buffer (50 µM Tris-HCl pH 8.0, 150 mM NaCl, 0.25% NP40). The desired amount of cell lysate was diluted in protein binding buffer (supplemented with 1 mM DTT and 1 mM PMSF) to 300 µL and incubated with beads at 4 °C for 16 h with rotation. Following washing one time with protein binding buffer, two times with TBS-T (20 mM Tris-HCl pH 7.6, 150 mM NaCl, 0.02% Tween 20), and two times with DPBS, the beads were resuspended in 15 µL DPBS and 5 µL 4x SDS sample buffer (200 mM Tris-HCl pH 6.8, 8% SDS, 40% glycerol, 0.08% bromophenol blue, 4% β-mercaptoethanol) and subsequently denatured at 95 °C for 5 min.

Sodium dodecyl sulfate-polyacrylamide gel electrophoresis (SDS-PAGE) and Western blot were performed to quantify the interaction between hMBD1 proteins and the VEGF-A probes. In short, the samples were loaded onto the 8% SDS-PAGE gel and the electrophoresis was conducted with 120 V for 70 min. After blotting onto the 0.2 µM PVDF membrane with the Trans-Blot Turbo transfer kit (Bio-rad, #1704272), the C-terminally Myc-tagged hMBD1 constructs were detected by the rabbit monoclonal Myc-Tag antibody 71D10 (1:1000 dilution, Cell Signaling, #2278) and the Alexa Fluor 750-conjugated goat anti-rabbit secondary antibody (1:1000 dilution, Invitrogen, # A-21039). Additionally, beta-tubulin from the cell lysate was detected by the mouse anti-beta tubulin primary antibody (1:1000 dilution, Cell Signaling, #86298) and the Dylight680-conjugated goat anti-mouse secondary antibody (1:5000 dilution, Invitrogen, #10797775). The Odyssey® DLx imaging system (LI-COR) was used to detect immunofluorescence.

Uncaging 1 in solution

Deprotection of **1** was carried out independently in simple buffer to illustrate the uncaging efficiency. 100 µL of 0.5 mM **1** in DPBS was irradiated in black 96-well plate with flat polymer coverslip bottom (ibidi, 89626) by the microscope light source mentioned above (violet LED with 395/25 nm bandpass filter at 100% intensity, Lumencor SPECTRA X light engine®). Irradiation of each well was performed by multiple image alignment (MIA) function to acquire panoramic well picture using a 10x objective. Every image in the MIA scanning area was acquired with the desired exposure time ranging from 10 to 500 ms. The UV-Vis absorption spectrum was then obtained by a NanoDrop 2000 (Thermo Fisher Scientific, v1.6).

SUPPLEMENTARY TABLES

Table S1. Oligonucleotides for plasmids construction.

Name	Sequence (5'→3')
o3167_ShP	CGTTCCGGACTACGCTTCTGAGCAGAAGCTGATCTCAGAGGAGGACCTGTGAATCGGTAGGAATTCGCGGCCG
o3257_TzL	tgaacctccgccacctgaccctccaccgccggtaccTACAAATGTGTTGTAAGGC
o3290_TzL	tctGAGCAGAAGCTGATCTCAG
o3291_TzL	GGCGCGCCCAACTTTGCG
o3292_TzL	aacgcaaagttgggcgcgccATGGCTGAGGACTGGCTG
o3293_TzL	cgccggtaccgttaatCTGCTTTCTAGCTCCAGGTTTTTTAAG
o3294_TzL	tagaaagcagattaacggtaccggcggtggagggtcaggtggcggaggttcaGTGAGCAAGGGCGAGGAG
o3295_TzL	tctgagatcagcttctgctcagaCTTGTACAGCTCGTCCATGC
o3387_ShP	GGAGGTGGCGGTGGCAGCGGG
o3388_ShP	CGCGCCCGCTGCCACCGCCACCTCCGC
o3642_TzL	CTGGctagCGCCATGCCGAAAAAGAAACGC
o3643_TzL	GCGTTTCTTTTTCGGCATGGCGctagCCAG
o3730_TzL	GAAGCGCCGCGAAGTCTTTTGTAAGTCAGGGGCCACCTGTG
o3731_TzL	CACAGGTGGCCCCTGACTTACAAAAGACTTCGCGGCGCTTC
o3732_TzL	CACAGGAGACAGGATCTGCAGCAAAGTTGAGCTGACTCG
o3733_TzL	CGAGTCAGCTCAACTTTGCTGCAGATCCTGTCTCCTGTG
o3756_TzL	GACAGGATCCGATAGAAAGTTGAGCTGACTCGATAC
o3757_TzL	GAGTCAGCTCAACTTTCTATCGGATCCTGTCTCCTGTG
o4128_TzL	AGGAGACAGGATCGCCTAGAAAGTTGAGCTGACTCGATAC
o4129_TzL	GAGTCAGCTCAACTTTCTAGGCGATCCTGTCTCCTGTGGG
o4302_BiR	CCTGGCTGCCCTAGCAAGAGGTCCAAAGACCTTAAAAAACCTGGAGC
o4305_BiR	GTTTTTTAAGGTCTTTGGACCTCTTGCTAGGGCAGCCAGG
o4479_TzL	AGAACCGCAAGGCCGGGGCCGCAGCAGCCTGCCTACGG
o4480_TzL	TAGGCAGGCTGCTGCGGCCCCGGCCTTGCGGTTCTGCC
o4558_TzL	TCTGAGCGTCCACACTAGGCCCCTGACTTGCGAAAG
o4592_TzL	AAGTCAGGGGCCTAGTGTGGACGCTCAGACACCTATTAC
o4594_TzL	AAGTCAGGGGCCaGCTGTGGACGCTCAGACACCTATTAC
o4560_TzL	TCTGAGCGTCCACAGCTGGCCCCTGACTTGCGAAAG

Table S2. Protein coding sequences used in this study.

Protein	Sequence
hMBD1	MAEDWLDCPALGPGWKRREVFRKSGATCGRSDTYYQSPTGDRIRSKVELTRYLGPACDLT<br>LFDFKQGILCYPAPKAHPVAVASKKRKKPSRPAKTRKRQVGPQSGEVRKEAPRDETKADT<br>DTAPASFPAPGCCENCGISFSGDGTQRQRLKTLCKDCRAQRIAFNREQRMFKRVGCGECA<br>ACQVTEDCGACSTCLLQLPHDVASGLFCKCERRRCLRIVERSRGCGVCRGCQTQEDCGHC<br>PICLRPPRPGLRRQWKCVQRRCLRGKHARRKGGCDSKMAARRRPGAQPLPPPPPSQSPEP<br>TEPHPRALAPSPPAEFIYYCVDEDELQPYTNRRQNRKCGACAACLRRMDCGRCDFCCDKP<br>KFGGSNQKRQKCRWRQCLQFAMKRLLPSVWSESEDGAGSPPPYRRRKRPSSARRHHLGPT<br>LKPTLATRTAQPDHTQAPTKQEAGGGFVLPPPGTDLVFLREGASSPVQVPGPVAASTEAL<br>LQEAQCSGLSWVVALPQVKQEKADTQDEWTPGTAVLTSPVLVPGCPSKAVDPGLPSVKQE<br>PPDPEEDKEENKDDSASKLAPEEEAGGAGTPVITEIFSLGGTRFRDTAVWLPRSKDLKKP<br>GARKQ
hMBD1-ΔTRD	MAEDWLDCPALGPGWKRREVFRKSGATCGRSDTYYQSPTGDRIRSKVELTRYLGPACDLT<br>LFDFKQGILCYPAPKAHPVAVASKKRKKPSRPAKTRKRQVGPQSGEVRKEAPRDETKADT<br>DTAPASFPAPGCCENCGISFSGDGTQRQRLKTLCKDCRAQRIAFNREQRMFKRVGCGECA<br>ACQVTEDCGACSTCLLQLPHDVASGLFCKCERRRCLRIVERSRGCGVCRGCQTQEDCGHC<br>PICLRPPRPGLRRQWKCVQRRCLRGKHARRKGGCDSKMAARRRPGAQPLPPPPPSQSPEP<br>TEPHPRALAPSPPAEFIYYCVDEDELQPYTNRRQNRKCGACAACLRRMDCGRCDFCCDKP<br>KFGGSNQKRQKCRWRQCLQFAMKRLLPSVWSESEDGAGSPPPYRRRKRPSSARRHHLGPT<br>LKPTLATRTAQPDHTQAPTKQEAGGGFVLPPPGTDLVFLREGASSPVQVPGPVAASTEAL<br>LQEAQCSGLSWVVALPQVKQEKADTQDEWTPGTAVLTSPVLVPGCPSKRSKDLKKPGARK<br>Q
hMBD1-<br>R22C+R44C	MAEDWLDCPALGPGWKRREVFCKSGATCGRSDTYYQSPTGDRICSKVELTRYLGPACDLT<br>LFDFKQGILCYPAPKAHPVAVASKKRKKPSRPAKTRKRQVGPQSGEVRKEAPRDETKADT<br>DTAPASFPAPGCCENCGISFSGDGTQRQRLKTLCKDCRAQRIAFNREQRMFKRVGCGECA<br>ACQVTEDCGACSTCLLQLPHDVASGLFCKCERRRCLRIVERSRGCGVCRGCQTQEDCGHC<br>PICLRPPRPGLRRQWKCVQRRCLRGKHARRKGGCDSKMAARRRPGAQPLPPPPPSQSPEP<br>TEPHPRALAPSPPAEFIYYCVDEDELQPYTNRRQNRKCGACAACLRRMDCGRCDFCCDKP<br>KFGGSNQKRQKCRWRQCLQFAMKRLLPSVWSESEDGAGSPPPYRRRKRPSSARRHHLGPT<br>LKPTLATRTAQPDHTQAPTKQEAGGGFVLPPPGTDLVFLREGASSPVQVPGPVAASTEAL<br>LQEAQCSGLSWVVALPQVKQEKADTQDEWTPGTAVLTSPVLVPGCPSKAVDPGLPSVKQE<br>PPDPEEDKEENKDDSASKLAPEEEAGGAGTPVITEIFSLGGTRFRDTAVWLPRSKDLKKP<br>GARKQ
hMBD1-<br>C338A+C341A	MAEDWLDCPALGPGWKRREVFRKSGATCGRSDTYYQSPTGDRIRSKVELTRYLGPACDLT<br>LFDFKQGILCYPAPKAHPVAVASKKRKKPSRPAKTRKRQVGPQSGEVRKEAPRDETKADT<br>DTAPASFPAPGCCENCGISFSGDGTQRQRLKTLCKDCRAQRIAFNREQRMFKRVGCGECA<br>ACQVTEDCGACSTCLLQLPHDVASGLFCKCERRRCLRIVERSRGCGVCRGCQTQEDCGHC<br>PICLRPPRPGLRRQWKCVQRRCLRGKHARRKGGCDSKMAARRRPGAQPLPPPPPSQSPEP<br>TEPHPRALAPSPPAEFIYYCVDEDELQPYTNRRQNRKAGAAAACLRRMDCGRCDFCCDKP<br>KFGGSNQKRQKCRWRQCLQFAMKRLLPSVWSESEDGAGSPPPYRRRKRPSSARRHHLGPT<br>LKPTLATRTAQPDHTQAPTKQEAGGGFVLPPPGTDLVFLREGASSPVQVPGPVAASTEAL

	LQEAQCSGLSWVVALPQVKQEKADTQDEWTPGTAVLTSPVLVPGCPSKAVDPGLPSVKQE PPDPEEDKEENKDDSASKLAPEEEAGGAGTPVITEIFSLGGTRFRDTAVWLPRSKDLKKP GARKQ

Table S3. Oligonucleotides for protein-DNA interaction assay.

Name	Sequence (5'→3')
o4681_LeE	(5′-Biotinylated)TTTCCAAAGCCCATTCCCT
o4666_LeE	AGTGACCCCTGGCCT
o5488_LeE (VEGF-A template)	TTTCCAAAGCCCATTCCCTCTTTAGCCAGAGCCGGGGTGTGCAGACGGCAGTCACTAGGGG GCGCTCGGCCACCACAGGGAAGCTGGGTGAATGGAGCGAGCAGCGTCTTCGAGAGTGAGGA CGTGTGTGTCTGTGTGGGTGAGTGAGTGTGTGCGTGTGGGGTTGAGGGCGTTGGAGCGGGG AGAAGGCCAGGGGTCACT

Table S4. Colocalization analysis with Pearson's correlation coefficient for Figure 2.

Figure	Channel 1	Channel 2	R value
2c	MBD1 wt	Dapi	0.91
2c	MBD1 R22C+R44C	Dapi	0.46
2c	MBD1 T27S	Dapi	0.8
2e (Before light)	MBD1 S45 → **1**	SiR-DNA	0.2
2e (20 min after light)	MBD1 S45 → **1**	SiR-DNA	0.78
2f (Before light)	MBD1 T27 → **1**	SiR-DNA	0.59
2f (20 min after light)	MBD1 T27 → **1**	SiR-DNA	0.55
2g (Before light)	MBD1 S45 → **1**	SiR-DNA	0.34
2g (20 min after light)	MBD1 S45 → **1**	SiR-DNA	0.85
2g (Before light)	MBD1 R22C+R44C+S45 → **1**	SiR-DNA	0.22
2g (20 min after light)	MBD1 R22C+R44C+S45 → **1**	SiR-DNA	0.26
2h (Before light)	MBD1 S45 → **1**	SiR-DNA	0.12
2h (20 min after light)	MBD1 S45 → **1**	SiR-DNA	0.43
2h (Before light)	MBD1 R22C+R44C+S45 → **1**	SiR-DNA	0.1
2h (20 min after light)	MBD1 R22C+R44C+S45 → **1**	SiR-DNA	0.07

Table S5. Foci number analysis for Figure 2.

Figure	Construct	Foci number
2c	MBD1 wt	28
2c	MBD1 R22C+R44C	10
2c	MBD1 T27S	34
2e (Before light)	MBD1 S45 → **1**	21
2e (20 min after light)	MBD1 S45 → **1**	47
2f (Before light)	MBD1 T27 → **1**	31

2f (20 min after light)	MBD1 T27 → **1**	30
2g (Before light)	MBD1 S45 → **1**	5
2g (20 min after light)	MBD1 S45 → **1**	31
2g (Before light)	MBD1 R22C+R44C+S45 → **1**	4
2g (20 min after light)	MBD1 R22C+R44C+S45 → **1**	4
2h (Before light)	MBD1 S45 → **1**	8
2h (20 min after light)	MBD1 S45 → **1**	26
2h (Before light)	MBD1 R22C+R44C+S45 → **1**	7
2h (20 min after light)	MBD1 R22C+R44C+S45 → **1**	8

SUPPLEMENTARY REFERENCES

[1] S. Palei, B. Buchmuller, J. Wolffgramm, Á. Muñoz-Lopez, S. Jung, P. Czodrowski, D. Summerer, *J Am Chem Soc* **2020**, *142*, 7289–7294.

[2] T.-C. Lin, S. Palei, D. Summerer, *ACS Chem Biol* **2022**, *17*, 1844–1852.

[3] N. Le Meur, F. Hahne, B. Ellis, *FlowCore: Data Structures Package for Flow Cytometry Data*, **2007**.

[4] K. Lo, F. Hahne, R. R. Brinkman, R. Gottardo, *BMC Bioinformatics* **2009**, *10*, 1–8.

[5] K. Lo, R. R. Brinkman, R. Gottardo, in *Cytometry Part A*, John Wiley & Sons, Ltd, **2008**, pp. 321–332.

[6] M. Malek, M. J. Taghiyar, L. Chong, G. Finak, R. Gottardo, R. R. Brinkman, *Bioinformatics* **2015**, *31*, 606–607.

[7] Hahne F, Gopalakrishnan N, Khodabakhshi AH, Wong C, Lee K, **2022**.

[8] V. Phu, W. Jiang, R. Gottardo, G. Finak, *Bioinformatics* **2018**, *34*, 3951–3953.

[9] Brocher Jan, **2022**.

[10] J. Schindelin, I. Arganda-Carreras, E. Frise, V. Kaynig, M. Longair, T. Pietzsch, S. Preibisch, C. Rueden, S. Saalfeld, B. Schmid, J.-Y. Tinevez, D. J. White, V. Hartenstein, K. Eliceiri, P. Tomancak, A. Cardona, *Nat Methods* **2012**, *9*, 676–682.

[11] Lindahl, Abraham, Hess, van der Spoel, **2020**, DOI 10.5281/zenodo.3685919.

[12] I. Ohki, N. Shimotake, N. Fujita, J.-G. Jee, T. Ikegami, M. Nakao, M. Shirakawa, *Cell* **2001**, *105*, 487–497.

[13] M. V. Shapovalov, R. L. Dunbrack, *Structure* **2011**, *19*, 844.

[14] E. F. Pettersen, T. D. Goddard, C. C. Huang, G. S. Couch, D. M. Greenblatt, E. C. Meng, T. E. Ferrin, *J Comput Chem* **2004**, *25*, 1605–1612.

[15] A. Croitoru, S. J. Park, A. Kumar, J. Lee, W. Im, A. D. Mackerell, A. Aleksandrov, *J Chem Theory Comput* **2021**, *17*, 3554–3570.

[16] K. Vanommeslaeghe, E. Hatcher, C. Acharya, S. Kundu, S. Zhong, J. Shim, E. Darian, O. Guvench, P. Lopes, I. Vorobyov, A. D. Mackerell, *J Comput Chem* **2010**, *31*, 671–690.

[17] W. Yu, X. He, K. Vanommeslaeghe, A. D. MacKerell, *J Comput Chem* **2012**, *33*, 2451–2468.

[18] K. Vanommeslaeghe, E. P. Raman, A. D. MacKerell, *J Chem Inf Model* **2012**, *52*, 3155–3168.

[19] K. Vanommeslaeghe, A. D. MacKerell, *J Chem Inf Model* **2012**, *52*, 3144–3154.

[20] T. D. Goddard, C. C. Huang, E. C. Meng, E. F. Pettersen, G. S. Couch, J. H. Morris, T. E. Ferrin, *Protein Science* **2018**, *27*, 14–25.

[21] E. F. Pettersen, T. D. Goddard, C. C. Huang, E. C. Meng, G. S. Couch, T. I. Croll, J. H. Morris, T. E. Ferrin, *Protein Science* **2021**, *30*, 70–82.
